# A Comprehensive Survey of Tools and Software for Active Subnetwork Identification

**DOI:** 10.3389/fgene.2019.00155

**Published:** 2019-03-05

**Authors:** Hung Nguyen, Sangam Shrestha, Duc Tran, Adib Shafi, Sorin Draghici, Tin Nguyen

**Affiliations:** ^1^Department of Computer Science and Engineering, University of Nevada, Reno, NV, United States; ^2^Department of Computer Science, Wayne State University, Detroit, MI, United States; ^3^Department of Obstetrics and Gynecology, Wayne State University, Detroit, MI, United States

**Keywords:** active module, active subnetwork, subnetwork identification, data integration, PPI network, network analysis

## Abstract

A recent focus of computational biology has been to integrate the complementary information available in molecular profiles as well as in multiple network databases in order to identify connected regions that show significant changes under different conditions. This allows for capturing dynamic and condition-specific mechanisms of the underlying phenomena and disease stages. Here we review 22 such integrative approaches for active module identification published over the last decade. This article only focuses on tools that are currently available for use and are well-maintained. We compare these methods focusing on their primary features, integrative abilities, network structures, mathematical models, and implementations. We also provide real-world scenarios in which these methods have been successfully applied, as well as highlight outstanding challenges in the field that remain to be addressed. The main objective of this review is to help potential users and researchers to choose the best method that is suitable for their data and analysis purpose.

## 1. Introduction

From human society to cellular activity, collaborative interactions, i.e., small units working in concert to accomplish certain functions, are an essential part of life. In complex multicellular organisms, their survival and health depend on the integrated activity of billions or trillions of cells organized into organ systems. Even in a single cell, the smallest structural and biological unit of life, fundamental processes, from DNA replication and energy production, to intercellular and intracellular signaling, often involve multiple biochemical reactions and molecular interactions taking place at multiple levels (transcriptomics, epigenomics etc.).

In order to have a good understanding of cellular functions at the systems-level, one needs to correctly identify and interpret all functional interactions of DNA, RNA, and proteins of organisms of interest (Szklarczyk et al., 2010). In turn, this has lead to the development of knowledge bases of functional modules and large networks of intermolecular interactions and pathways. Biological networks, which are graphical representation of genes, proteins, DNAs, RNAs, or even small miRNAs and their functional interactions, are rapidly accumulated in public databases, including HPRD (Keshava Prasad et al., 2008), DIP (Salwinski et al., 2004), KEGG (Kanehisa et al., 2017), Reactome (Croft et al., 2014), and many other curated interactome networks developed for human and model species (Harbison et al., 2004; Stelzl et al., 2005; Yu et al., 2008; Ravasi et al., 2010). Many computational approaches have been developed to mine such interactome networks in order to better understand cellular processes and disease mechanisms (Spirin and Mirny, 2003). Topological modules (Girvan and Newman, 2002), within which nodes are well-connected and the interactions are more concentrated compared with those outside, are among the most intensively studied research areas. However, as functional interactions are annotated in static experimental conditions, network databases alone fail to account for the dynamic nature of biological systems and thus fail to provide a full representation of cellular interactions.

Recently, with the advancement of high-throughput technologies, biological data of different kinds have rapidly accumulated in public repositories. Taken alone, molecular data only represents a snapshot of biological systems and often fail to elucidate biological mechanisms. When projected onto biological networks, however, molecular profiles and expression changes have the potential to reflect the perturbation of complex cellular network and thus allow for comprehensive monitoring of biological systems (Cowen et al., 2017; Yi et al., 2017). A recent focus of computational biology has been to integrate the complementary information available in molecular profiles as well as in multiple network databases in order to identify active modules, i.e., well-connected subnetworks that are significantly perturbed under different conditions (Mitra et al., 2013). These approaches have been widely applied and proven to be powerful in elucidating biological mechanisms of underlying physiological and disease phenotypes (Chuang et al., 2007; Bapat et al., 2010; Qiu et al., 2010; Zhang and Ouellette, 2011; Shafi et al., 2019).

In this document, we categorize and review 22 such subnetwork identification methods based on the following criteria: their availability and user interface, the type of input the method requires, subnetwork seeding and construction, and statistical approaches used to assess the significance of the identified subnetworks. We classify these approaches into six different categories according to the techniques used to traverse the global network in order to construct the active subnetworks. In section 2, we discuss the availability, implementation, types of experimental input and reference network databases that the surveyed methods use. In section 3, we categorize and compare the methods according to the way they traverse and expand the subnetwork. In section 4, we include real-world scenarios in which the surveyed methods were successfully applied. In section 5, we discuss the limitations of current knowledge bases and outstanding challenges in method development. In section 6, we systematically recapitulate the 22 approaches by highlighting their key characteristics and differences. We also provide detailed descriptions for individual methods in Supplementary Material.

To the best of our knowledge, this is the first article that provides such in-depth discussion and covers a large number of tools for active subnetwork identification. A recent survey of biological networks (Mitra et al., 2013) discussed active network identification, among other topics, and provided a list of tools. However, this article covers many topics and its wide breadth means there was some limitation in the depth to which these tools could be covered. In addition, many of the tools listed there are outdated and/or not maintained anymore. More recently, another survey (He et al., 2017) focused on assessing the performance of 10 subnetwork analysis methods using simulations. This survey, however, provides even fewer details and discussion of each individual method. In contrast, here we provide a comprehensive review of a total of 22 methods for active subnetwork identification, highlighting their availability, implementation, applicable network databases, underlying mathematical and algorithmic principles, as well as advantages and limitations for each method. The main objective of this review is to help potential users and researchers to choose methods that are suitable for their data and analysis purpose.

## 2. Software and Databases

### 2.1. Availability and Implementation

Table 1 shows the 22 methods we review in this article. Although more computational methods for subnetwork identification have been published, we only review methods that are associated with executable packages that can actually be used by people other than the authors. This table provides the following information about each tool: (i) their availability (link to the tool), (ii) implementation (standalone package, web interface, user interface, programming language), (iii) reference to the original articles, (iv) citations, and (v) software license. We believe that these details are crucial for users to know before spending a significant amount of time to understand the software and perform analyses.

**Table 1:** Computational tools for active subnetwork identification.

**Method**	**Availability**	**Web**	**Pkg**	**Code**	**GUI**	**Cml**	**References**	**Cit**.	**Cit./year**	**Lic**.
**GREEDY ALGORITHM**										
DIAMOnD	https://github.com/barabasilab/DIAMOnD/	✗	✓	Python	✗	✓	Ghiassian et al., 2015	74	24	**free
GXNA	http://statweb.stanford.edu/%7eserban/gxna/	✗	✓	C++	✗	✓	Nacu et al., 2007	141	12	free
MATISSE	http://acgt.cs.tau.ac.il/matisse/	✗	✓	Java	✓	✗	Ulitsky and Shamir, 2007	313	28	free
CEZANNE	http://acgt.cs.tau.ac.il/matisse	✗	✓	Java	✓	✗	Ulitsky and Shamir, 2009	111	12	free
PinnacleZ	http://apps.cytoscape.org/apps/pinnaclez	✗	✓	Java	✓	✗	Chuang et al., 2007	1,414	128	free
RME Module Detection	http://brl.bcm.tmc.edu/rme/index.rhtml	✗	✓	Ruby	✗	✓	Miller et al., 2011	82	11	MIT
**EVOLUTIONARY ALGORITHM**										
BMRF-Net	https://sourceforge.net/projects/bmrfcjava/	✗	✓	C++, Java	✓	✗	Shi et al., 2015	4	1	free
COSINE	https://cran.r-project.org/package=COSINE	✗	✓	R	✗	✓	Ma et al., 2011	64	9	GPL-3
GLADIATOR	http://www.cs.tau.ac.il/~roded/GLADIATOR.zip	✗	✓	Python	✗	✓	Silberberg et al., 2017	3	3	free
jActiveModules	http://apps.cytoscape.org/apps/jactivemodules	✗	✓	Java	✓	✗	Ideker et al., 2002	1,115	69	GPL
MOEA	https://github.com/WeiqiChen/MOEA-active-module-identification	✗	✓	MATLAB	✗	✓	Chen et al., 2017	1	1	free
**DIFFUSION-FLOW EMULATION MODEL**										
BioNet & Hienz	http://www.mi.fu-berlin.de/w/LiSA/Heinz	✗	✓	R, Python	✗	✓	Dittrich et al., 2008	175	17	MIT
							Beisser et al., 2010	413	51	
HotNet & HotNet2	http://compbio.cs.brown.edu/projects/hotnet2/	✗	✓	Python, MATLAB	✗	✓	Vandin et al., 2011	237	33	**free
							Leiserson et al., 2015	313	104	
RegMod	https://bmcbioinformatics.biomedcentral.com/articles/ 10.1186/1471-2105-11-26	✗	✓	MATLAB	✗	✓	Qiu et al., 2010	73	9	free
ResponseNet	http://netbio.bgu.ac.il/respnet/	✓	✗	Python	✓	✗	Lan et al., 2011	26	free
							Basha et al., 2013	57	8	
								15	3	
TimeXNet	http://timexnet.hgc.jp/	✓	✓	Java	✓	✓	Patil and Nakai, 2014	6	1	**free
**RANDOM WALK ALGORITHM**										
EnrichNet	http://lcsb-enrichnet.uni.lu/enrichnet/index.php	✓	✗	R, PHP	✓	✗	Glaab et al., 2012	171	28	**free
Walktrap-GM	https://github.com/petrochilos/walktrap-GM/	✗	✓	R	✗	✓	Petrochilos et al., 2013	20	4	GPL
**MAXIMAL CLIQUE IDENTIFICATION**										
MEMo	http://sanderlab.org/tools/memo.html	✗	✓	Java, Python	✗	✓	Ciriello et al., 2012	377	62	LGPL
ModuleDiscoverer	https://www.leibniz-hki.de/en/modulediscoverer.html	✗	✓	R	✗	✓	Vlaic et al., 2018	3	3	GPL
**CLUSTERING-BASED METHOD**										
ClustEx	http://bioinfo.au.tsinghua.edu.cn/member/jgu/clustex/	✗	✓	C++	✗	✓	Gu et al., 2010	54	6	free
SAMBA	http://acgt.cs.tau.ac.il/samba/	✗	✓	Java	✓	✓	Tanay et al., 2004	431	30	free

*Availability provides links to software. Web and Pkg indicate whether the web interface and the standalone package are available, respectively. Code lists the programming languages that were used to implement the software. GUI and Cml indicate whether the tool has GUI and command line, respectively. Reference provides the corresponding publication. Cit. shows the total number of citations while Cit/year shows the number of citations per year. License provides software license; GPL is an abbreviation for the GNU General Public License; LGPL stands for GNU Lesser General Public License; MIT stands for Massachusetts Institute of Technology*;

** *free is free for academic and non-commercial use. Note that PinnacleZ is not compatible with Cytoscape 3 but it can still be downloaded from web archive https://web.archive.org/web/20120105141450/http://chianti.ucsd.edu/cyto_web/plugins/pluginjardownload.php?id=170*.

One often thinks that the strengths of a computational approach mostly depend on its algorithmic novelty and time and space complexity. However, the availability and implementation of the software have become more and more important (Nguyen et al., 2018). Since there are many tools available in the market, if a method is not well-implemented, potential users will simply pick another tool that is ready-to-run. It is unlikely that life scientists, who are the intended audience of these software, invest time to learn a programming language in order to implement complex algorithms reported in some papers. Practically, input and output format, graphical user interface, programming language, user-friendliness, and documentation are all important factors to be considered. More importantly, since reproducibility has become an outstanding issue recently, software availability and version control are critical for quality control and for reproducing the results reported in scientific papers (Sandve et al., 2013). For that reason, many journals today require authors to make their code available before publishing the paper.

At the time of this review, all of the 22 surveyed methods are available as either a standalone package or a web-based tool. Among these, there are 20 standalone packages and three web-based tools (one tool has both standalone package and web-based tool). Standalone tools are more often chosen to make use of the computational power of users' local machines or servers. Some of these packages provide a friendly interface for users to interact with. These software usually provides interactive features for users to manipulate the network and explore the data, which is illustrative and convenient. Some of them, e.g., PinnacleZ, BMRF-Net, and jActiveModules, are distributed as plugins of Cytoscape (Shannon et al., 2003) to make use of its friendly interactive interface in manipulating networks. The rest provide command line interface or APIs for users to conduct experiments. Users usually need a third-party software to visualize the result networks such as Cytoscape. An advantage of tools with a command line interface is that it is easier for advanced users to integrate and embed these tools in their automated analysis pipeline. Most standalone tools require some administrative skills to install. Since these tools require interactome data, users are expected to download, locally store, and periodically update the network databases (partial or full copy). A standalone tool usually does not require internet access to perform analysis, which enhances the security and privacy of the experimental data.

Web-based tools (ResponseNet, TimeXNet, and EnrichNet), on the other hand, rely on a remote server to conduct analysis and provide computational power and a graphical interface; therefore, a local installation is generally not needed. Web-based tools are more user-friendly than standalone tools; however, they require an internet connection and a browser for access. In terms of cybersecurity and data privacy, this is considered a disadvantage compared to standalone tools. One major advantage of web-based tools is that most updates are transparent. In turn, this enhances the users' performance and enables collaboration between users by eliminating the burden of local installation and the need to keep it up-to-date.

The choice of the programming language used for the implementation also influences how well the method will be received. Tools that are well-implemented and packaged are more accepted than those that are poorly implemented or not user-friendly. Many methods implemented in Java provide good performance, can run on multiple platforms (Windows, Linux, MacOS), and offer a nice interactive user interface. For packages providing command line or APIs, it is worth to mention that the programming language plays a vital role in attracting users. For example, R users will prefer using an R package rather than learning a new language (such as Python or MATLAB). The programming language can also be an obstacle when there is a need to integrate a tool written in a different language to the current analysis pipeline. Most tools published as R packages can be easily installed due to R's user-friendly package manager. Other standalone tools written in C++, Python, and Ruby provide a command line interface to execute the analysis. Tools implemented in C++ also need to be compiled before using.

We also report the number of citations (and citations per year) for each method according to Google Scholar. Although the number of Google citation is not the right metric to assess a method's novelty or performance, it partially reflects how well a tool is accepted or known among researchers in the community. Finally, we report the license of each software. All of the surveyed software are free-of-charge for academic purposes. Many of them are freely available for non-academic users as well.

### 2.2. Experimental Data and Network Databases

Table 2 shows the input of each method, as well as the corresponding network databases and applicable species. Up to date, most methods are designed for analyzing human diseases using protein-protein interactions. Among the 22 methods, only six were designed to work with other species, including *Rattus norvegicus* (ModuleDiscoverer), *Mus musculus* (MATISSE, CEZANNE, TimeXNet), *Saccharomyces cerevisiae* (MATISSE, CEZANNE, jActiveModule, ResponseNet, TimeXNet, SAMBA), *Drosophila melanogaster* (MATISSE, CEZANNE), and *C. elegans* (MATISSE, CEZANNE). Most methods claim to be able to work with other species provided that the interaction network is available.

**Table 2:** Active module identification approaches along with their corresponding input, network databases and species.

**Method**	**Experimental input**	**MC**	**Network database**	**Species**
**GREEDY ALGORITHM**				
DIAMOnD	Gene expression	✗	TRANSFAC, IntAct, MINT, BioGRID, HPRD, CORUM, PhosphositePlus, curated network (Vinayagam et al., 2011)	*Homo sapiens*
GXNA	Gene expression	✗	EntrezGene, KEGG	*Homo sapiens*
MATISSE & CEZANNE	Gene expression	✗	SGD, BioGRID, BIND, HPRD, GO, MIPS, KEGG	*Homo sapiens, Mus musculus, Saccharomyces cerevisiae, Drosophila melanogaster, Caenorhabditis elegans*
PinnacleZ	Gene expression	✓	GO, Cell Circuits	*Homo sapiens*
RME Module Detection	Somatic mutation (SNP, CNV)	✗	–	*Homo sapiens*
**EVOLUTIONARY ALGORITHM**				
BMRF-Net	Gene expression	✗	HPRD	*Homo sapiens*
COSINE	Gene expression	✓	HPRD	Homo sapiens
GLADIATOR	Multiple lists of proteins where each list is associated with a disease	✓	Curated Network (Menche et al., 2015)	*Homo sapiens*
jActiveModules	Gene expression	✓	BIND, TRANSFAC, GAL	*Saccharomyces cerevisiae*
MOEA	Gene expression	✗	BioGRID, KEGG, GO	*Homo sapiens*
**DIFFUSION-FLOW EMULATION MODEL**				
BioNet & Hienz	Gene expression& survival information	✗	HPRD	*Homo sapiens*
HotNet & HotNet2	Mutation frequency of genes	✗	KEGG, HPRD	*Homo sapiens*
RegMod	Gene expression	✗	HPRD, MSigDB, GO	*Homo sapiens*
ResponseNet	Weighted list of DE genes and proteins	✗	Curated Network (Yeger-Lotem et al., 2009)	*Saccharomyces cerevisiae*
TimeXNet	Three list of DE genes and log fold change at initial intermediate and late stages	✗	HitPredict, InnateDB, TRANSFAC, KEGG	*Mus musculus, Saccharomyces cerevisiae*
**RANDOM WALK ALGORITHM**				
EnrichNet	A list of genes or proteins	✗	STRING, KEGG, BioCarta, WikiPathways, Reactome, InterPro, NCI-PID	*Homo sapiens*
Walktrap-GM	Gene expression	✗	HPRD, KEGG	*Homo sapiens*
**MAXIMAL CLIQUE IDENTIFICATION**				
MEMo	Gene expression, somatic mutation, copy number variation	✗	Reactome, MSKCC Cancer Cell Map, NCI-PID, HPRD, GO, PANTHER, INOH	*Homo sapiens*
ModuleDiscoverer	Gene expression	✗	STRING	*Rattus norvegicus*
**CLUSTERING-BASED METHOD**				
ClustEx	DE genes and fold change	✗	HPRD	*Homo sapiens*
SAMBA	Gene expression, transcription factor (TF) binding	✗	MIPS	*Saccharomyces cerevisiae*

*Experimental Input indicates various input requirements that need to be supplied for the method to function. MC indicates whether the tool can handle multiple conditions (i.e., multiple diseases instead of the typical disease vs. control). Network Database provides network interaction information for various kinds of methods. Species gives information about species whose data could be used by the computational tools*.

A subnetwork detection analysis typically requires two different kinds of input: (1) experimental data, and (2) interactome networks. Experimental data is generally data obtained from high-throughput technologies, such as gene and protein expression, somatic mutation, and copy number alteration. Among the 22 methods, only BioNet & Heinz uses the survival information to score genes in addition to differential analysis of expression data (Supplementary section 1.12). Most methods are designed for comparative analysis of two phenotypes, e.g., condition (disease) vs. control (healthy). Among the 22 methods, only four methods can detect subnetworks that are perturbed across multiple diseases or conditions. These are PinnacleZ, COSINE, GLADIATOR, and jActiveModules. These methods use statistics and tests (e.g., *F*-test, mutual information) that are able to compare more than two groups of samples in order to score the candidate subnetworks (see section 6 for discussions).

Different analysis methods use different input formats. There are only three methods that use mutation profiles as input, including RME Module Detection, HotNet, and MEMo. These methods aim to identify subnetworks that have more genes with mutations than expected. Most other methods accept the whole gene expression matrix, in which rows represent genes and columns represent samples from different phenotypes. Some methods accept only differentially expressed (DE) genes/proteins and their corresponding statistics (fold-change, *p*-value). TimeXNet is the only method that requires time-course data in the format of DE genes. The list of DE genes or proteins can be obtained by using a predefined cut-off based on *p*-value, fold-change, or both. Network approaches relying on input DE genes, however, might be overly sensitive to both selection method and cut-off threshold. First, a slight change in the threshold can greatly alter the list of DE genes (Nam and Kim, 2008). Second, different selection methods often produce different lists of DE genes. For example, the list of genes with high fold-changes is often not identical to the list of genes with significant *p*-values. In addition, for the same disease, independent studies or measurements often produce inconsistent sets of DE genes (Tan et al., 2003; Ein-Dor et al., 2005, 2006). This makes network analysis methods that rely on DE gene list even less reliable.

The input format for each method can be different depending on the programming language and the implementation of the method. For R packages, a common input of gene expression profiles is usually a matrix object where rows represent samples and columns represent genes (or vice versa). The input network can be passed as an adjacency matrix representing the relationship between nodes. For Cytoscape's plugins, the input network is in the format of Cytoscape network input files. With other methods, gene expression and network data are usually stored in flat files with a specific format defined by the software.

Besides experimental data, most methods also require interactome data or biological networks for their analysis. Biological networks are graphs representing either protein-protein networks or gene-gene networks. In these networks, the nodes are proteins or genes, and edges are interactions between them. Each network can contain additional information such as directions of the interactions (in directed networks), weights of nodes and edges, and other knowledge about the proteins, genes or their interactions (e.g., different types of interactions). RME Module Detection is the only method that does not require a predefined network as input. It constructs the global network from mutation data before extracting active subnetworks.

Among the network databases presented in Table 2, many of them are widely used in pathway analysis such as KEGG, HPRD, STRING, Reactome, NCI-PID, and BioGRID. In the subnetwork analysis, while most methods use a single protein network database to conduct the experiment, some methods, such as jActiveModules, MOEA, MEMo, TimeXNet, and DIAMOnD, combine multiple networks from different sources to construct a larger network. Since overlap among network databases is small (Chaurasia et al., 2006; Prieto and De Las Rivas, 2006), combining multiple databases can potentially increase the knowledge about interactome networks to build a more comprehensive biological network.

## 3. Methods

**Figure 1:**
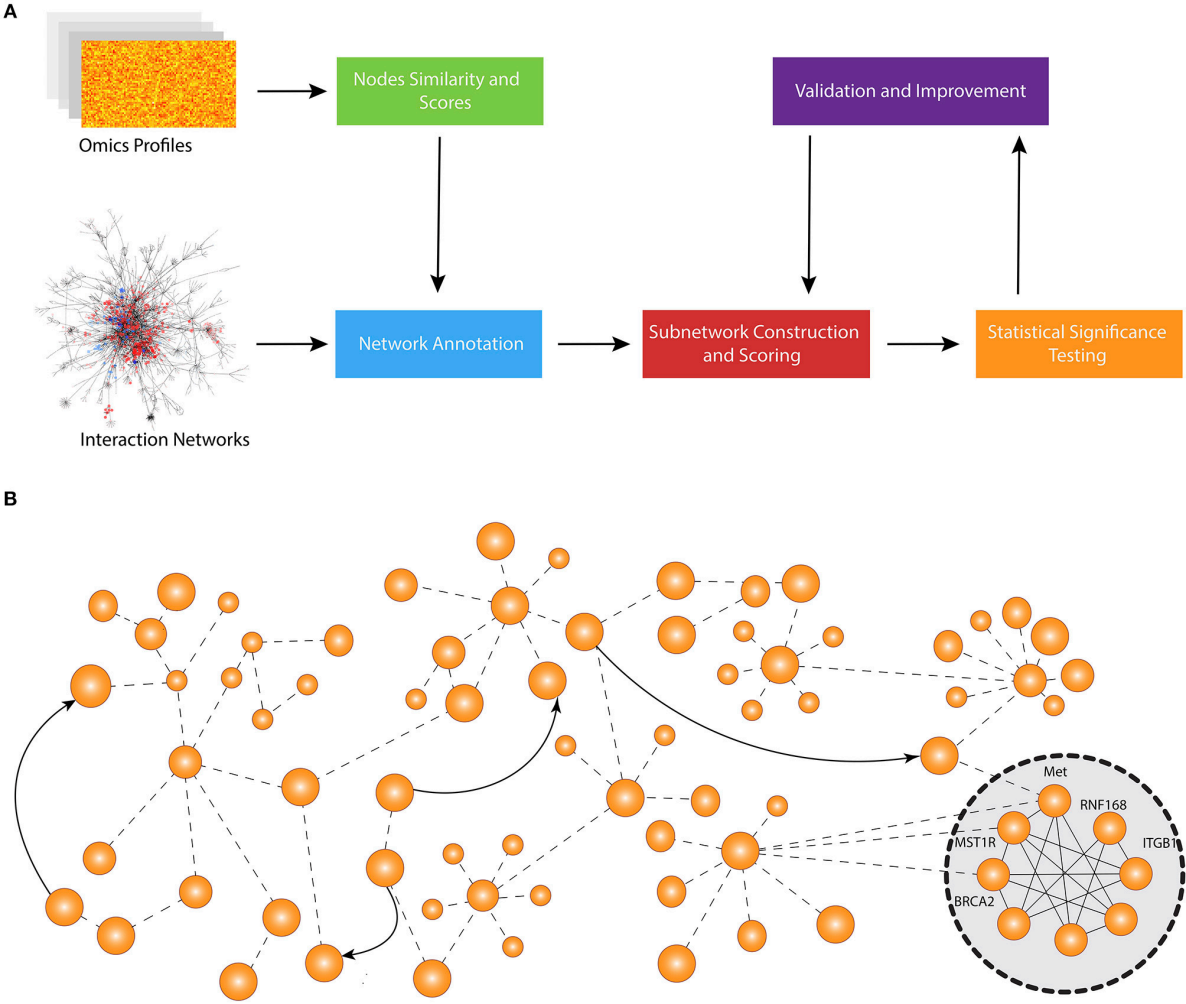
Overall workflow of active subnetwork identification. **(A)** Schematic representation of computational approaches that integrate molecular profiles with known interactions accumulated in knowledge bases. Most methods start by scoring the nodes and calculating node similarity that reflects the expression change (e.g., between disease and control) and correlation between genes, respectively. Then, they adjust the scores and edge weights by taking into consideration the topological order and interaction between genes and proteins. The next step is to construct the subnetworks using edge weights and node scores. Typically, each method develops a specific subnetwork extension strategy in order to optimize a specific subnetwork scoring function using node scores and edge weights. After the subnetworks are constructed, each method performs a hypothesis testing to assess the statistical significance of identified modules. Some methods also repeatedly reconstruct the subnetwork after statistical tests to find a more optimal solution. **(B)** An example network and identified active subnetwork. The subnetwork are often a very well-connected component of the global network.

Figure 1 shows the schematic representation of computational approaches that integrate phenotypic molecular profiles with known interactions accumulated in network databases. Most methods start by scoring the nodes and calculate node similarity that reflects the expression change (e.g., between disease and control) and the correlation between genes, respectively. Then, they adjust the scores and edge weights by taking into consideration the topological order and interaction between genes and proteins. The next step is to construct the subnetworks using edge weights and node scores. Typically, each method deploys its own subnetwork extension strategy in order to optimize a particular subnetwork scoring function using node scores and edge weights. After the subnetworks are constructed, each method performs a hypothesis testing to assess the statistical significance of identified modules. Some methods also repeatedly reconstruct the subnetwork after the statistical tests in order to find a more optimal solution.

Here we divide the methods into 6 categories according to the way the subnetworks are expanded: (1) greedy algorithms, (2) evolutionary algorithms, (3) maximal clique identification, (4) random walk algorithms, (5) diffusion emulation models, and (6) clustering-based methods. We summarize the methods in each category, providing the big picture and insights. Section 6 contains a detailed characteristics of each method.

### 3.1. Greedy Algorithms

In this section, we review six approaches that utilize a greedy strategy in order to construct active subnetworks, including GXNA (Gene eXpression Network Analysis), CEZANNE (Co-Expression Zone ANalysis using NEtworks), MATISSE (Module Analysis via Topology of Interactions and Similarity SEts), DIAMOnD (DIseAse MOdule Detection), PinnacleZ, and RME (recurrent and mutually exclusive) Module Detection.

The common flow of greedy algorithms consists of three major steps: (i) seed nodes selection (ii) subnetwork expansion, and (iii) significance testing. In the first step, the seeds can be randomly selected nodes (GXNA and PinnacleZ), high-scoring nodes (MATISSE and CEZANNE), user-defined nodes (DIAMOND and GXNA), or all nodes in the network (RME Module Detection). In the second step, each method then greedily extends the seeds with neighboring nodes with the objective to maximize the subnetwork's score. The procedure is repeated until further expansion does not increase the objective function. Some methods introduce early stopping criteria, such as the maximum size (RME Module Detection) or the improvement rate (PinnacleZ). In the third step, the statistical significance of the identified subnetworks is assessed by comparing its score against the scores obtained from random subnetworks (CEZANNE, PinnacleZ, RME Module Detection), or from permuting sample and gene labels (GXNA, MATISSE, PinnacleZ). This statistical significance of a subnetwork represents the probability of observing such score or higher, just by chance. The smaller the *p*-value, the less likely that such extreme score is observed by chance, i.e., the more likely the subnetwork has significant changes or significantly perturbed under the impact of the disease. DIAMOnD is the only method in this category that does not assess the statistical significance of the resulted subnetworks.

Greedy algorithms are fast and intuitive. However, since the decision at each step aims to improve the current state of the solution without paying attention to the global situation, it does not guarantee to produce the most optimal path. In fact, there is a high chance that the greedy algorithm does not find the global optima. Therefore, the selection of starting points plays a vital role in identifying optimal solutions. In addition, since this approach depends heavily on maximizing the score of the network by repeatedly adding adjacent nodes, the scoring function plays a vital role in the entire analysis, affecting the construction as well as the statistical significance of the obtained subnetworks. The methods scoring the network based on the similarity or correlation in gene expression change (MATISSE, CEZANNE, RME Module Detection, and PinnacleZ) tend to expand the modules to contain only highly similar genes, which can result in subnetworks missing important intermediate genes. Moreover, in some cases, these methods can produce large subnetworks with hundreds of genes that are difficult to interpret.

### 3.2. Evolutionary Algorithms

Here we review five approaches that use evolutionary algorithms to search for active modules with optimal scores: BMRF-Net (Bagging Markov Random Field), COSINE (COndition SpecIfic sub-NEtwork), GLADIATOR (GLobal Approach for DIsease AssociaTed mOdule Reconstruction), jActiveModule, and MOEA (Multi-Objective Evolutionary Algorithm). Similar to greedy approaches, evolutionary methods first define a scoring formula for each node and each edge as well as for a subnetwork whose score is often a weighted aggregation of nodes and edges belonging to the subnetwork. Each tool then uses either Generic Algorithm (COSINE and MOEA) or Simulated Annealing (BMRF-Net, GLADIATOR, and jActiveModules) to search for an optimal subnetwork with the highest aggregate score. Among the five methods, only BMRF-Net and jActiveModule access the statistical significance of the obtained subnetwork using resampling and bootstrap, respectively.

Abstractly, the subnetwork construction can be formulated as a global optimization problem. Given *p* as the total number of genes, a subnetwork is represented as a binary vector of length *p*. The *i*^*th*^ element in the vector being 1 means that the *i*^*th*^ gene is present in the network. Evolutionary algorithms seek to find a binary vector that optimizes a certain scoring function. Simulated Annealing (SA) algorithm initializes a subnetwork by assigning each node as either active or inactive with a probability (default $\frac{1}{2}$). At each iteration, the algorithm randomly chooses a node and toggle the node's state (from active to inactive and vice versa). It then recalculates the aggregate score of the subnetwork. If the new score is greater than the old score, the state of the node is kept toggled. Otherwise, the node is kept toggled with a certain probability (to avoid being trapped in a local minimum). The algorithm returns the highest scoring subgraph after a number of iterations. Note that GLADIATOR maximizes the similarity (using Jaccard index) between the connected modules provided for different diseases instead of optimizing the aggregate score of nodes and edges. The classical simulated annealing algorithm gets its inspiration from heat treatment in metallurgy which involves annealing metal to increase crystal size while reducing defects (Kirkpatrick et al., 1983).

Genetic Algorithms (SA), on the other hand, are inspired by natural selection, the process that drives biological evolution. The algorithm initialization sets certain genes (e.g., nodes with high scores) to 1 (active) and considers these genes as the starting population. Individuals in the population (parents) are then selected in pairs for reproduction based on their fitness score, in which crossover and mutation are happening. Crossover involves exchanging information from the parents to produce offspring while random mutations (with a low probability) alter the offspring to maintain diversity. The algorithm stops when the population has converged.

Although both GA and SA produce good quality solutions in the problem of finding optimal subnetworks, there is always a trade-off between running time and solution quality, which is affected by the size of the solution in GA and the temperature decay rate in SA (Adewole et al., 2012). The advantage of these algorithms is that they are not limited to the size and the complexity of the search space. Therefore, it can work with very large networks. In contrast to greedy algorithms, genetic algorithms aim to find the global solution and have proven to be very efficient in finding an approximation of global optima. Since GA and SA are both efficient in solving the problem of finding optimal subnetworks, it is important that the scoring process reflects precisely the perturbation and signal propagation of the subnetworks.

### 3.3. Diffusion-Flow Emulation Models

In this section, we discuss five methods that emulate diffusion flow phenomena in order to construct active subnetworks. Two of these are inspired by the heat diffusion process (HotNet and RegMod), while three others by the water flow phenomenon (BioNet & Heinz, ResponseNet, and TimeXNet). These are methods that aim to find a global solution through algorithmic optimization. Among the five, only TimeXNet and HotNet provide a statistical significance of the obtained active modules by using a permutation test.

Given a weighted and directed protein-protein interaction (PPI) network, BioNet & Heinz, ResponseNet, and TimeXNet emulate an abstract flow from a source node to a sink node through capacity- and cost-associated edges. The objective is to minimize the total cost from a source node to a sink node through a linear formulation in which variables represent the flow over each edge. Each edge of the network is assigned with: (i) a cost that is inversely proportional to the interaction reliability between the two connected nodes, and (ii) a flow capacity that is proportional to the similarity in molecular measurements of the two connected genes. The optimization problem is then solved using constrained linear programming, in which constraints (linear equalities or inequalities) are given to nodes and edges. While ResponseNet and TimeXNet produce only one optimal solution, Heinz allows users to explore different sub-optimal networks by adding a hamming distance to the optimal subnetwork to constrain the differences of the returned sub-optimal networks.

Heat diffusion algorithms, HotNet and RegMod, define the problem of finding active subnetworks as a heat diffusion model. Given a PPI network in which nodes weight represents initial heat, the optimization process delivers heat through edges until the heat in the network is equilibrium. Hot subnetworks are constructed after selecting edges transferring a total heat amount larger than a certain threshold. RegMod uses a heat diffusion kernel to calculate the similarity between two nodes, then computes the score for each gene that represents its relationship with other genes in the network. Active subnetworks are obtained by extracting high scoring genes.

### 3.4. Random Walk Algorithms

A random walk is a simulated path consisting of successive random transitions through a mathematical space, for example, an integer set or a 2-D plane. The transitions are not necessarily a complete random action but rather can be biased toward a specific direction. In a biological network, the connections (or interaction intensities) between different pairs of proteins are different. When applying the random walk algorithm on the network, the walk is more likely to stay in the subnetwork with high interactions among the members, because the chance of the walk choose the lower interaction paths to escape the subnetwork is small. The performance of the algorithm is heavily affected by the method used to weight the interactions.

Walktrap-GM (R package) and EnrichNet (web interface) are the two tools that utilize random walks to identify active subnetworks. EnrichNet requires a list of starting proteins while Walktrap-GM uses as input gene expression data. To build the weighted interaction network, Walktrap-GM calculates the weight of each edge as the average fold-change of the two connected nodes. In contrast, ErinchNet uses the weight extracted from STRING 9.0 database, which could be argued to be better, as it is combined confidence from different sources such as curated databases, gene co-occurrence, text mining, etc. To travel in the network, Walktrap-GM uses the random walk algorithm which transits from the current node to its adjacent nodes with a probability based on the weight of the linking edge and the degree of the current node. Using the transition probabilities, the distance between two nodes and between two communities formed by the random walk process are then calculated. The traverse will merge two communities if it minimizes the mean of the squared distances between each node and its community. On the other hand, EnrichNet uses a random walk with restart to emphasize the importance of starting nodes. Therefore, the result would be subnetworks that has strong connections with the input gene list. Overall, Walktrap-GM is expected to be more useful to look for new active modules while EnrichNet is expected to perform better in the deeper investigation of already-known modules. Walktrap-GM also assesses the statistical significance of the obtained subnetworks using bootstrap.

### 3.5. Maximal Cliques Identification

This class of methods for active subnetwork identification is focusing on finding cliques, i.e., subnetworks in which every node is connected with all others. This approach is based on the assumption that all the proteins in an active module would have tight connections with the rest. Due to the lack of efficient algorithms to find these cliques in a dense network (Tomita et al., 2006), a preprocessing step to simplify the network is necessary. Two methods in this review (MEMo and ModuleDiscover) have different solutions to this problem. ModuleDiscover tries to filter out the interactions that are not strong enough based on the data from STRING database. In contrast, MEMo applies three different kinds of filters based on significantly mutated gene, copy number regions of interest and mRNA expression to retain only the altered genes in their network. Then, cliques are extracted from these filtered networks.

The advantage of these methods is the reliability of the identified subnetworks, due to the nature of clique (strong interaction in subnetwork). Moreover, by modifying the algorithm, various kinds of data could be applied to the simplification step to refine the network even more before the identification of active modules. As a potential disadvantage, ModuleDiscover's reliance on the prior knowledge in the STRING database means that the discovery of new modules is essentially impossible. Also for MEMo, the aggressive filtering (three filter layers) means that some important information may be lost in the process.

### 3.6. Clustering-Based Methods

In this section, we review two methods using different approaches in the identification of active modules from other groups. These are ClustEx, which is based on a hierarchical clustering algorithm, and SAMBA, which uses biclustering on a bipartite graph. ClustEx first calculates weights and distances for the edges using the Pearson correlation of the expression of the genes associated with the nodes. Subsequently, ClustEx clusters the genes using hierarchical clustering. It then identifies the active modules through two steps. In the first step, it looks for node pairs with the distance below a given threshold and assigns the connecting path as the initial clusters. In the second step, it expands the initial cluster to the surrounding genes. Finally, the nodes that are visited by the 10-shortest path in this expanded cluster are identified as belonging to an active module. As potential limitations, one can note that during the first step, ClustEx calculates the distances between every pair of nodes which could be a heavy computational task. Moreover, due to the nature of the expanding process, which is determined by the 10-shortest path, some important nodes in a tightly connected cluster could be left out.

Unlike other methods, SAMBA takes a completely different approach to identify the active modules. Instead of building one single interaction network using genes as nodes, they build a weighted bipartite graph where nodes on one side represent the genes and nodes on the other side represent properties of proteins encoded by them. The connection between two parts represents the probability for a gene to have a specific property. The locally optimal subgraphs are identified using biclustering and overlapping is minimized by limiting the shared properties between subgraphs. The performance of this model is heavily dependent on the selections of properties layer, which could make it challenging to apply SAMBA to a new disease.

## 4. Applications

The 22 surveyed methods have been widely applied in real-world scenarios to find disease gene signatures, dysfunctional pathways, common mechanisms of different diseases, as well as to discover drug and toxicity effects on different organisms. PinnacleZ, despite being the most highly cited method, was mostly cited for the discovery reported in the paper. The method jActiveModules appears to be the most used tool for discovery and understanding biological mechanisms. As a Cytoscape plugin, jActiveModules has its advantages in network visualization and manipulation. At the time of this survey, we found approximately 80 studies that utilized this software. BioNET as an R package, was also applied in real-world settings in more than 30 studies. Other tools including EnrichNet, MEMo, and MATISSE were utilized in more than 10 studies. The number of studies and manuscript DOIs are reported in Supplementary section 2.

PPI networks have been widely used in identifying disease biomarker and sample classification. For example, Chuang et al. (2007) used PinnacleZ to classify patients with breast cancer and Yuan et al. (2017) applied jActiveModules to find gene signatures for leukemia patients for sample stratification purposes. Network-based signatures have proven to be more reliable and reproducible than signatures identified from gene expression data alone. In fact, proteins involved in cancers tend to show a high level of connectivity in the PPI networks (Jonsson and Bates, 2006). Therefore, discovering gene signatures for specific diseases can be greatly improved by identifying significantly impacted subnetworks from the PPI network, especially when the known disease genes are highlighted as seed genes in the network (Shafi et al., 2019).

Active subnetwork approaches have also been utilized to discover dysfunctional pathways of diseases. For example, Skov et al. (2012) used jActiveModules to study biological pathways and networks that are dysregulated in type 2 diabetes. The study by Riazuddin et al. (2017) made use of MATISSE to find novel gene candidates for the biological pathways of intellectual disability disorder. Similarly, Sharma et al. (2015) identified key pathways within the asthma module discovered by the DIAMOnD method. Resulted modules from subnetwork methods provide meaningful insights to dysfunctional processes of the underlying disease phenotypes.

The discovered subnetworks, although are resulted from a specific disease, can also be used to predict disease-causing genes for similar diseases or other complex diseases (Oti and Brunner, 2007). By identifying responsive modules corresponding to certain diseases, the associations among diseases can be discovered by network similarity. For example, Dong et al. (2014) used jActiveModules to extract active modules of type 2 diabetes and coronary heart disease to find pathways that are important to both diseases. In another study, Wuchty et al. (2010) used significant subnetworks discovered by PinnacleZ to find pathways that help to discriminate major glioma subtypes. Studying associations between dysfunctional modules from different phenotypes may reveal the true mechanism of complex diseases such as cancers.

Drug and toxicity studies have also made use of active subnetwork identification to discover their effects on different organisms. For example, jActiveModules was used to identify the network regions that is active under methamphetamine (Bortell et al., 2017) and dioxin (Alexeyenko et al., 2010) exposure. Similarly, BioNet was applied to extract top-scoring networks to understand the impact of drug combinations on lymphoma disease (Zhao et al., 2014). BioNet was also used to find candidates for drug targets (Cursons et al., 2015). Although drug targets are typically regarded as single proteins, most drugs often interact with a larger number of proteins. By studying drug-response subnetworks, the overall effects of a drug can be revealed not only the efficacy of the drug to the target proteins but also its side effects.

## 5. Challenges in Subnetwork Identification

Even though subnetwork identification methods have been applied in many real-world applications, there are challenges that have not been addressed. In this section, we highlight the limitations of existing knowledge bases, as well as identify outstanding challenges from the method development perspectives.

### 5.1. Knowledge Bases

One major challenge is that most PPI knowledge bases are incomplete. For example, widely used networks in HPRD and BioGRID cover at most 50% of the known human PPIs (De Las Rivas and Fontanillo, 2010). In consequences, analysis results using these knowledge bases may be incomplete due to possible omissions of important factors. Another example is that the number of genes in KEGG remained around 5,000 over the past few years whereas the number of protein-coding genes is estimated to be between 19,000 and 20,000 (Ezkurdia et al., 2014). Integrative methods using networks and gene expression data are forced to work on a much reduced gene space. In many cases, using the reduced number of features in a classification algorithm decreases the classification performance (Staiger et al., 2013), suggesting that some important features (genes) had been left out by the PPI networks. One approach to increase the coverage of the PPI networks is to combine multiple knowledge bases to build a more comprehensive biological network.

Another important limitation of existing knowledge bases is that they are unable to keep up with the high-resolution information that has become available with the advancement of high-throughput technologies and multi-omics assays. For example, data obtained from RNA-Seq experiments allows us to identify transcripts that are active under certain conditions. Multiple transcripts mapping to the same gene can have distinct or even opposite functions due to the alternative splicing (Wang et al., 2008). Although this information is crucial to reveal the underlying biological mechanisms, the majority of the PPI knowledge bases only provides information at the gene level. In addition, knowledge bases do not provide information regarding cell types, conditions and time points each of which is essential to reveal the true phenomenon of a given biological condition. Finally, existing knowledge bases offer at most limited options to integrate multi-omics data. In the past decade, molecular data of all kinds, from transcriptomics to genomics, epigenetics, and non-coding RNA have accumulated on public repositories with unprecedented rates. However, most subnetwork approaches are limited to gene/protein data. A great wealth of these data remain unused since knowledge bases mostly store information about protein or gene interactions. Future approaches need to develop graphical models that take into consideration changes at different levels (e.g., methylation, miRNA, mRNA) to exploit the complementary information available in different types of omics data.

### 5.2. Method Development

One key challenge for subnetwork method development is the lack of universally accepted gold-standard to validate the identified subnetwork modules. Computational approaches are typically assessed by simulated data or by well-studied biological datasets (He et al., 2017; Vlaic et al., 2018). The advantage of using simulation data is that the ground truth is always known. Thus it can be used to compare different methods using sensitivity and specificity. However, simulation is often oversimplified and unable to capture the complexity of living organisms. On the other hand, when using real biological data, the biology is never fully known. In addition, many papers presenting new analysis methods include results obtained from only a couple of datasets and researchers are often influenced by the observer-expectancy effect (Sackett, 1979). Designing benchmark real datasets where the true mechanisms are known would help address this issue.

Furthermore, the majority of the active subnetwork identification methods do not account for the complexity of protein interactions. Most of active subnetwork identification methods and network clustering approaches, produce only non-overlap modules. These methods were developed based on the assumption that a protein can be active in at most one module. However, it is known that most proteins may involve in many biological processes. In addition, a disease can go through different stages and a protein may take place in different active modules at different time points. Producing large networks containing all possible interactions is insufficient to reveal the underlying mechanisms for complex diseases. Reporting different networks for different stages of dynamic networks, in this case, will significantly help to interpret the signal of the disease.

Finally, *p*-value-based approaches are subject to potential bias under the null hypothesis. In principle, the null distribution is used to assess the significance of the observed result obtained from an experiment. The *p*-values obtained from any sound statistical test are assumed to follow a uniform distribution (with the interval 0–1) (Fodor et al., 2007; Barton et al., 2013). Although this issue is yet to be investigated in the field of subnetwork identification, it has been shown that many computational methods for network and pathway analysis have a systematic bias toward pathways related to cancer and well-studied diseases (Nguyen et al., 2017). In the study, the authors created a large pool of healthy individuals and then randomly compare two groups of healthy people. Interestingly, the *p*-value distributions of cancer pathways are extremely biased toward zero and thus are found significant in many analyses that have nothing to do with cancer. Similarly, subnetwork analyses are expected to be biased toward well-studied diseases and network modules. To overcome this problem, *p*-values of the candidate modules should be calculated under the null to demonstrate that a method is not biased under the null.

## 6. Discussions

**Figure 2:**
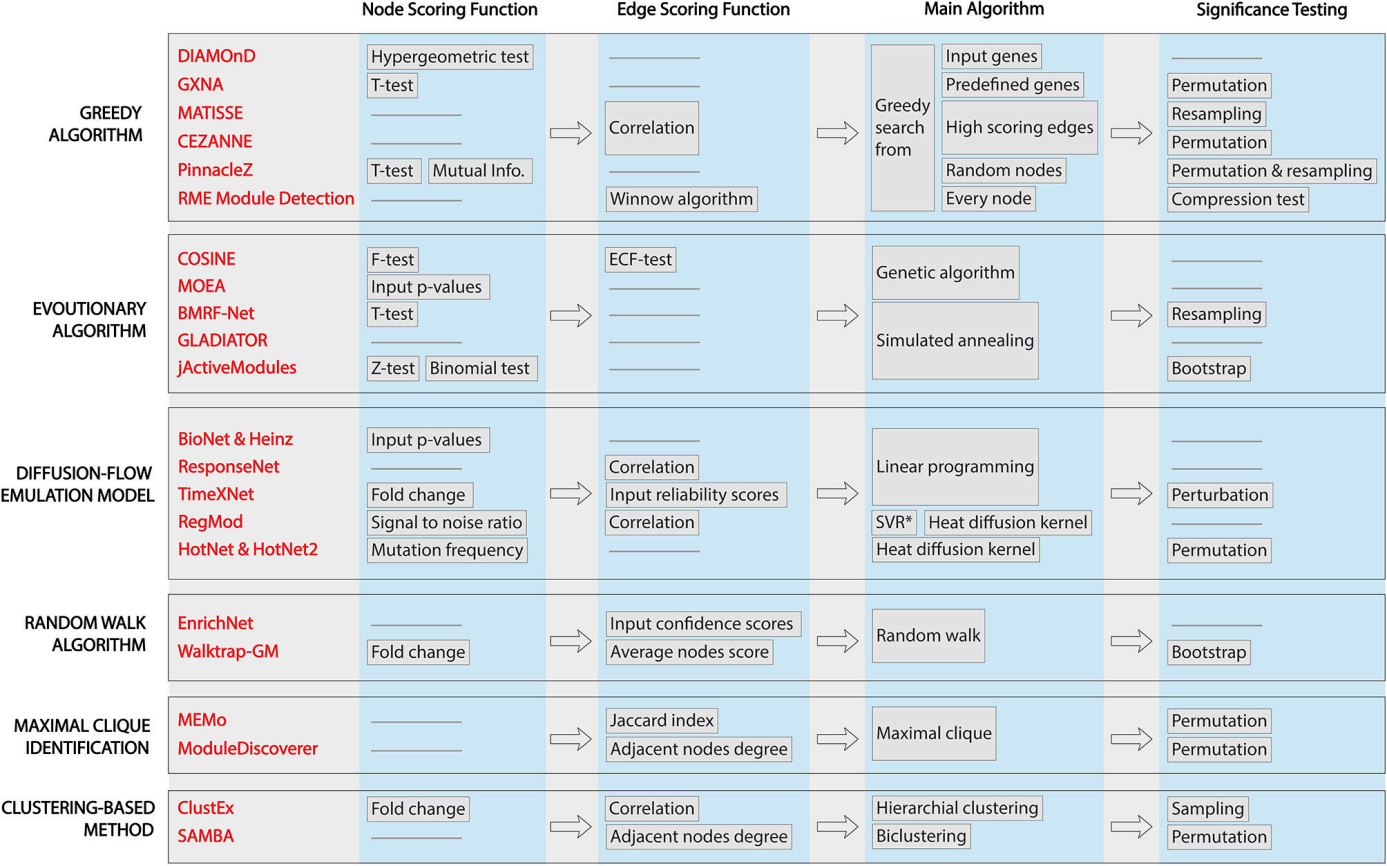
Workflows of active module identification approaches. The figure highlights the key characteristics and key differences between each method. From left to right are the techniques applied in each approach: (i) node scoring, (ii) edge scoring, (iii) algorithm used to construct the subnetworks, and (iv) statistical test for assessing the significance of the identified active subnetworks. We note that GLADIATOR does not score nodes nor edges but uses Jaccard Index between input gene sets (of different diseases) as the objective for its simulated annealing algorithm.

All of the methods surveyed here aim to identify one or several active subnetworks for one or several input datasets. However, they differ in their assumptions about the relationship among genes, protein, or both, which leads to different scoring functions and traversal strategies. Figure 2 shows the workflows of the 22 methods, highlighting their characteristics and differences. From left to right are the techniques applied in each approaches: (i) node scoring, (ii) edge scoring, (iii) algorithm used to construct the subnetworks, and (iv) statistical test for assessing the significance of the identified active subnetworks. Note that GLADIATOR does not score nodes nor edges but rather it uses the Jaccard Index between input gene sets (of different diseases) as the objective for its simulated annealing algorithm. In this review, we categorize the 22 approaches according to the way they construct their network (main algorithm).

The problem of finding optimal subnetworks with the highest network score is NP-hard. Therefore, many methods address this problem via a heuristic approach that does not guarantee a global optimum. Random walk and greedy algorithms construct their modules by initializing the seeds and greedily extend the modules. Therefore, the results obtained with these methods will depend on the choice of the seeds. In a large network of tens of thousands nodes, it is harder to find a seed that leads to the global optima. Diffusion-flow emulation models, on the other hand, model the problem as a mathematical optimization that aims to find the global optimum using algorithmic optimization. For example, maximum-flow algorithms assign flow capacity and flow cost to nodes and edges and then find the global optimum using constrained linear programming. These mathematical approaches guarantee to reach a global optimum. Similarly, evolutionary algorithms also aim to find the global optimum or at least an approximation of it. The algorithms allow for transitions to states with a lower score in order to avoid being trapped at a local maximum/minimum. In principle, with a large number of iterations, these algorithms are likely to find a global solution.

Maximal clique approaches and clustering-based methods are distinct from the rest in terms of their goals and objectives. Maximal clique methods do not aim to find connected nodes with the best score. Instead, they aim to find groups of genes that interact with one another (every pair of nodes in a clique has an edge between them). However, it is not necessarily true that all genes in a clique always take part in certain biological processes. In addition, these methods may miss intermediate genes or proteins that play important roles in connecting those cliques. The clustering-based methods, on the other hand, assume that co-expressed genes are all involved in the same cellular process (ClustEx) or there is a hierarchical structure in the biological network (SAMBA). Since clustering approaches aim to group high-similarity genes into the same cluster without paying attention to the size of each cluster, the output can be highly imbalanced, including extremely large subnetworks that fail to provide insights into the underlying mechanisms of the given phenotypes.

The methods surveyed here use a wide range of scoring functions to score the nodes and edges. Most of them (except GLADIATOR) provide a scoring function for nodes or edges, but only some of them take into account the scores of both nodes and edges. While node-based scoring approaches look at the significance of one gene or protein in the context of the whole network, edge-based scoring networks look at the strength of the relationship among protein or gene. Without paying attention to the weight of the relationship between proteins or genes, node-based scoring methods may produce subnetworks that have high scoring nodes but do not have a meaningful relationship between nodes. Also, the edge-based scoring network may produce subnetworks that contain highly similar genes but have low significance in the network. These resulted subnetworks, unfortunately, will be difficult to interpret. Methods that take into account the scores of both nodes and edges are likely to produce a more accurate active module.

Node and edge scoring functions are the building blocks of the subnetwork score. In principle, they should be the summary statistics that capture the network perturbation, signal propagation as well as the changes between different phenotypes. Since each test and score is based on a certain assumption, users need to check if the assumption of each test matches the property of their data. For instance, the *z*-test and *t*-test assume that the data follows a normal distribution, while methods using fold-change assume that the effect size is the most important factor to capture the difference between the two conditions. Note that fold-change is highly dependent on the background signal, i.e., a shift in the range will significantly change fold change (e.g., 101 compared to 103 vs. 1 compared to 3) (Drăghici, 2011). Furthermore, the *t*-test, *z*-test, and fold-change approaches can only compare two conditions, while the F-test, mutual information, and binomial test allow users to capture changes across multiple conditions. Some methods do not take into account the scores of the nodes but rather require the user to input a gene list or protein list (GLADIATOR, ResponseNet, EnrichNet, and ClustEx) as the significant gene set to serve as starting points of the algorithm. These methods can be sensitive to the input gene list, in which small changes in the list can dramatically affect the resulted subnetworks. In contrast to the variety of node scoring, the edge scoring functions mostly rely on the correlation between two adjacent nodes to indicate the similarity between nodes.

The significance test used in a particular tool is also an important factor to consider. An aggregated score calculated for a module represents the level of signal perturbation or the degree of change observed in the subnetwork between different conditions. Similar to fold-change or effect-size, this score can be either the result of real biological changes or just by chance due to random noise. One needs to assess whether the observed change represents real biological differences. Therefore, a significance assessment should be done to assess how likely the aggregated score is observed just by chance under the null hypothesis, i.e., due to noise and chance alone. DIAMOnD, COSINE, MOEA, Bionet & Heinz, ResponseNet, and EnrichNet output the subnetworks and aggregated scores without performing a significance assessment. Thus, it is totally up to users to interpret the identified subnetworks and their scores. The remaining methods perform a significance assessment and calculate a *p*-value for each resulted subnetwork. For methods that provide multiple active subnetworks, a correction for multiple comparisons should be performed. Users can determine whether each subnetwork is significantly impacted by comparing the *p*-values with a pre-defined threshold.

## 7. Conclusions

In the past decades, there have been great efforts to mine network databases for identifying condition-specific cellular processes. One successful strategy has been to integrate these networks with molecular data to identify active subnetworks or modules that are involved in condition-specific biological functions. In this article, we review 22 methods that identify active subnetworks by integrating molecular data (e.g., expression profiles, protein, mutation) with known biological interaction accumulated in knowledge bases and public repositories. At the time of preparing this article, all surveyed methods are available as either a working standalone package or through a web-based interface. We categorize the 22 methods into five different categories according to the way they construct and extend the subnetwork. We summarize the pros and cons of each approach and category, focusing on their distinguishing characteristics and mathematical models. Our main objective is to help potential users and life scientists to choose methods that are most suitable for their available data and analysis purpose. This review will also help computational scientists to identify shortcomings of existing approaches in order to develop new methods that address current limitations.

## Author Contributions

TN and HN drafted the text with contributions from all co-authors: SS, DT, AS, and SD. SD reviewed and edited the final version.

### Conflict of Interest Statement

The authors declare that the research was conducted in the absence of any commercial or financial relationships that could be construed as a potential conflict of interest.

## References

[B1] Adewole A. Otubamowo K. Egunjobi T. Ng K. (2012). A comparative study of simulated annealing and genetic algorithm for solving the travelling salesman problem. Int. J. Appl. Inform. Syst. 4, 6–12. 10.5120/ijais12-450678

[B2] Alexeyenko A. Wassenberg D. M. Lobenhofer E. K. Yen J. Linney E. Sonnhammer E. L.. (2010). Dynamic zebrafish interactome reveals transcriptional mechanisms of dioxin toxicity. PLoS ONE 5:e10465. 10.1371/journal.pone.0010465 20463971 PMC2864754

[B3] Bapat S. A. Krishnan A. Ghanate A. D. Kusumbe A. P. Kalra R. S. (2010). Gene expression: protein interaction systems network modeling identifies transformation-associated molecules and pathways in ovarian cancer. Cancer Res. 70, 0008–5472. 10.1158/0008-5472.CAN-10-0447 20530682

[B4] Barton S. J. Crozier S. R. Lillycrop K. A. Godfrey K. M. Inskip H. M. (2013). Correction of unexpected distributions of P values from analysis of whole genome arrays by rectifying violation of statistical assumptions. BMC Genomics 14:161. 10.1186/1471-2164-14-161 23496791 PMC3610227

[B5] Basha O. Tirman S. Eluk A. Yeger-Lotem E. (2013). ResponseNet2.0: revealing signaling and regulatory pathways connecting your proteins and genes–now with human data. Nucleic Acids Res. 41, W198–W203. 10.1093/nar/gkt532 23761447 PMC3692079

[B6] Beisser D. Klau G. W. Dandekar T. Muller T. Dittrich M. T. (2010). BioNet: an R-Package for the functional analysis of biological networks. Bioinformatics 26, 1129–1130. 10.1093/bioinformatics/btq089 20189939

[B7] Bortell N. Basova L. Semenova S. Fox H. S. Ravasi T. Marcondes M. C. G. (2017). Astrocyte-specific overexpressed gene signatures in response to methamphetamine exposure *in vitro*. J. Neuroinflammation 14:49. 10.1186/s12974-017-0825-6 28279172 PMC5345234

[B8] Chaurasia G. Iqbal Y. Hänig C. Herzel H. Wanker E. E. Futschik M. E. (2006). UniHI: an entry gate to the human protein interactome. Nucleic Acids Res. 35(Suppl. 1), D590–D594. 10.1093/nar/gkl817 17158159 PMC1781159

[B9] Chen W. Liu J. He S. (2017). Prior knowledge guided active modules identification: an integrated multi-objective approach. BMC Syst. Biol. 11:8. 10.1186/s12918-017-0388-2 28361699 PMC5374590

[B10] Chuang H.-Y. Lee E. Liu Y.-T. Lee D. Ideker T. (2007). Network-based classification of breast cancer metastasis. Mol. Syst. Biol. 3:140. 10.1038/msb4100180 17940530 PMC2063581

[B11] Ciriello G. Cerami E. Sander C. Schultz N. (2012). Mutual exclusivity analysis identifies oncogenic network modules. Genome Res. 22, 398–406. 10.1101/gr.125567.111 21908773 PMC3266046

[B12] Cowen L. Ideker T. Raphael B. J. Sharan R. (2017). Network propagation: a universal amplifier of genetic associations. Nat. Rev. Genet. 18, 551–562. 10.1038/nrg.2017.38 28607512

[B13] Croft D. Mundo A. F. Haw R. Milacic M. Weiser J. Wu G.. (2014). The Reactome pathway knowledgebase. Nucleic Acids Res. 42, D472–D477. 10.1093/nar/gkv1351 24243840 PMC3965010

[B14] Cursons J. Leuchowius K. J. Waltham M. Tomaskovic-Crook E. Foroutan M. Bracken C. P.. (2015). Stimulus-dependent differences in signalling regulate epithelial-mesenchymal plasticity and change the effects of drugs in breast cancer cell lines. Cell Commun. Signal. 13:26. 10.1186/s12964-015-0106-x 25975820 PMC4432969

[B15] De Las Rivas J. Fontanillo C. (2010). Protein–protein interactions essentials: key concepts to building and analyzing interactome networks. PLoS Comput. Biol. 6:e1000807. 10.1371/journal.pcbi.1000807 20589078 PMC2891586

[B16] Dittrich M. T. Klau G. W. Rosenwald A. Dandekar T. Müller T. (2008). Identifying functional modules in protein–protein interaction networks: an integrated exact approach. Bioinformatics 24, i223–i231. 10.1093/bioinformatics/btn161 18586718 PMC2718639

[B17] Dong C. Tang L. Liu Z. Bu S. Liu Q. Wang Q.. (2014). Landscape of the relationship between type 2 diabetes and coronary heart disease through an integrated gene network analysis. Gene 539, 30–36. 10.1016/j.gene.2014.02.001 24508273

[B18] Drăghici S. (2011). Statistics and Data Analysis for Microarrays Using R and Bioconductor. London, UK: Chapman and Hall; CRC Press.

[B19] Ein-Dor L. Kela I. Getz G. Givol D. Domany E. (2005). Outcome signature genes in breast cancer: is there a unique set? Bioinformatics 21, 171–178. 10.1093/bioinformatics/bth469 15308542

[B20] Ein-Dor L. Zuk O. Domany E. (2006). Thousands of samples are needed to generate a robust gene list for predicting outcome in cancer. Proc. Natl. Acad. Sci. U.S.A. 103, 5923–5928. 10.1073/pnas.0601231103 16585533 PMC1458674

[B21] Ezkurdia I. Juan D. Rodriguez J. M. Frankish A. Diekhans M. Harrow J.. (2014). Multiple evidence strands suggest that there may be as few as 19 000 human protein-coding genes. Hum. Mol. Genet. 23, 5866–5878. 10.1093/hmg/ddu309 24939910 PMC4204768

[B22] Fodor A. A. Tickle T. L. Richardson C. (2007). Towards the uniform distribution of null P values on Affymetrix microarrays. Genome Biol. 8:R69. 10.1186/gb-2007-8-5-r69 17472745 PMC1929139

[B23] Ghiassian S. D. Menche J. Barabasi A. L. (2015). A DIseAse MOdule Detection (DIAMOnD) algorithm derived from a systematic analysis of connectivity patterns of disease proteins in the human interactome. PLoS Comput. Biol. 11:e1004120. 10.1371/journal.pcbi.1004120 25853560 PMC4390154

[B24] Girvan M. Newman M. E. (2002). Community structure in social and biological networks. Proc. Natl. Acad. Sci. U.S.A. 99, 7821–7826. 10.1073/pnas.122653799 12060727 PMC122977

[B25] Glaab E. Baudot A. Krasnogor N. Schneider R. Valencia A. (2012). EnrichNet: network-based gene set enrichment analysis. Bioinformatics 28, i451–i457. 10.1093/bioinformatics/bts389 22962466 PMC3436816

[B26] Gu J. Chen Y. Li S. Li Y. (2010). Identification of responsive gene modules by network-based gene clustering and extending: application to inflammation and angiogenesis. BMC Syst. Biol. 4:47. 10.1186/1752-0509-4-47 20406493 PMC2873318

[B27] Harbison C. T. Gordon D. B. Lee T. I. Rinaldi N. J. Macisaac K. D. Danford T. W.. (2004). Transcriptional regulatory code of a eukaryotic genome. Nature 431:99. 10.1038/nature02800 15343339 PMC3006441

[B28] He H. Lin D. Zhang J. Wang Y.-p. Deng H.-w. (2017). Comparison of statistical methods for subnetwork detection in the integration of gene expression and protein interaction network. BMC Bioinformatics 18:149. 10.1186/s12859-017-1567-2 28253853 PMC5335754

[B29] Ideker T. Ozier O. Schwikowski B. Siegel A. F. (2002). Discovering regulatory and signaling circuits in molecular interaction networks. Bioinformatics 18, S233–S240. 10.1093/bioinformatics/18.suppl_1.S233 12169552

[B30] Jonsson P. F. Bates P. A. (2006). Global topological features of cancer proteins in the human interactome. Bioinformatics 22, 2291–2297. 10.1093/bioinformatics/btl390 16844706 PMC1865486

[B31] Kanehisa M. Furumichi M. Tanabe M. Sato Y. Morishima K. (2017). KEGG: new perspectives on genomes, pathways, diseases and drugs. Nucleic Acids Res. 45, D353–D361. 10.1093/nar/gkw1092 27899662 PMC5210567

[B32] Keshava Prasad T. Goel R. Kandasamy K. Keerthikumar S. Kumar S. Mathivanan S.. (2008). Human protein reference database–2009 update. Nucleic Acids Res. 37(Suppl. 1), D767–D772. 10.1093/nar/gkn892 18988627 PMC2686490

[B33] Kirkpatrick S. Gelatt C. D. Vecchi M. P. (1983). Optimization by simulated annealing. Science 220, 671–680. 10.1126/science.220.4598.671 17813860

[B34] Lan A. Smoly I. Y. Rapaport G. Lindquist S. Fraenkel E. Yeger-Lotem E. (2011). ResponseNet: revealing signaling and regulatory networks linking genetic and transcriptomic screening data. Nucleic Acids Res. 39(Suppl. 2), W424–W429. 10.1093/nar/gkr359 21576238 PMC3125767

[B35] Leiserson M. D. Vandin F. Wu H. T. Dobson J. R. Eldridge J. V. Thomas J. L.. (2015). Pan-cancer network analysis identifies combinations of rare somatic mutations across pathways and protein complexes. Nat. Genet. 47, 106–114. 10.1038/ng.3168 25501392 PMC4444046

[B36] Ma H. Schadt E. E. Kaplan L. M. Zhao H. (2011). COSINE: COndition-SpecIfic sub-NEtwork identification using a global optimization method. Bioinformatics 27, 1290–1298. 10.1093/bioinformatics/btr136 21414987 PMC3138081

[B37] Menche J. Sharma A. Kitsak M. Ghiassian S. D. Vidal M. Loscalzo J.. (2015). Uncovering disease-disease relationships through the incomplete interactome. Science 347:1257601. 10.1126/science.1257601 25700523 PMC4435741

[B38] Miller C. A. Settle S. H. Sulman E. P. Aldape K. D. Milosavljevic A. (2011). Discovering functional modules by identifying recurrent and mutually exclusive mutational patterns in tumors. BMC Med. Genomics 4:34. 10.1186/1755-8794-4-34 21489305 PMC3102606

[B39] Mitra K. Carvunis A.-R. Ramesh S. K. Ideker T. (2013). Integrative approaches for finding modular structure in biological networks. Nat. Rev. Genet. 14, 719–732. 10.1038/nrg3552 24045689 PMC3940161

[B40] Nacu Ş. Critchley-Thorne R. Lee P. Holmes S. (2007). Gene expression network analysis and applications to immunology. Bioinformatics 23, 850–858. 10.1093/bioinformatics/btm019 17267429

[B41] Nam D. Kim S.-Y. (2008). Gene-set approach for expression pattern analysis. Brief. Bioinform. 9, 189–197. 10.1093/bib/bbn001 18202032

[B42] Nguyen T. Mitrea C. Draghici S. (2018). Network-based approaches for pathway level analysis. Curr. Protoc. Bioinformatics 61, 8–25. 10.1002/cpbi.42 30040185

[B43] Nguyen T. Mitrea C. Tagett R. Draghici S. (2017). DANUBE: Data-driven meta-ANalysis using UnBiased Empirical distributions - applied to biological pathway analysis. Proc. IEEE 105, 496–515. 10.1109/JPROC.2015.2507119 29706661 PMC5919277

[B44] Oti M. Brunner H. G. (2007). The modular nature of genetic diseases. Clin. Genet. 71, 1–11. 10.1111/j.1399-0004.2006.00708.x 17204041

[B45] Patil A. Nakai K. (2014). TimeXNet: identifying active gene sub-networks using time-course gene expression profiles. BMC Syst. Biol. 8:S2. 10.1186/1752-0509-8-S4-S2 25522063 PMC4290689

[B46] Petrochilos D. Shojaie A. Gennari J. Abernethy N. (2013). Using random walks to identify cancer-associated modules in expression data. BioData Mining 6:17. 10.1186/1756-0381-6-17 24128261 PMC4015830

[B47] Prieto C. De Las Rivas J. (2006). APID: agile protein interaction DataAnalyzer. Nucleic Acids Res. 34(Suppl. 2), W298–W302. 10.1093/nar/gkl128 16845013 PMC1538863

[B48] Qiu Y. Q. Zhang S. Zhang X. S. Chen L. (2010). Detecting disease associated modules and prioritizing active genes based on high throughput data. BMC Bioinformatics 11:26. 10.1186/1471-2105-11-26 20070902 PMC2825224

[B49] Ravasi T. Suzuki H. Cannistraci C. V. Katayama S. Bajic V. B. Tan K.. (2010). An atlas of combinatorial transcriptional regulation in mouse and man. Cell 140, 744–752. 10.1016/j.cell.2010.01.044 20211142 PMC2836267

[B50] Riazuddin S. Hussain M. Razzaq A. Iqbal Z. Shahzad M. Polla D.. (2017). Exome sequencing of pakistani consanguineous families identifies 30 novel candidate genes for recessive intellectual disability. Mol. Psychiatry 22, 1604–1614. 10.1038/mp.2016.109 27457812 PMC5658665

[B51] Sackett D. L. (1979). Bias in analytic research. J. Chron. Dis. 32, 51–63. 447779 10.1016/0021-9681(79)90012-2

[B52] Salwinski L. Miller C. S. Smith A. J. Pettit F. K. Bowie J. U. Eisenberg D. (2004). The database of interacting proteins: 2004 update. Nucleic Acids Res. 32(Suppl. 1), D449–D451. 10.1093/nar/gkh086 14681454 PMC308820

[B53] Sandve G. K. Nekrutenko A. Taylor J. Hovig E. (2013). Ten simple rules for reproducible computational research. PLoS Comput. Biol. 9:e1003285. 10.1371/journal.pcbi.1003285 24204232 PMC3812051

[B54] Shafi A. Nguyen T. Peyvandipour A. Nguyen H. Draghici S. (2019). A multi-cohort and multi-omics meta-analysis framework to identify network-based gene signatures. Front. Genet. 10:159 10.3389/fgene.2019.00159 PMC6434849 30941158

[B55] Shannon P. Markiel A. Ozier O. Baliga N. S. Wang J. T. Ramage D.. (2003). Cytoscape: a software environment for integrated models of biomolecular interaction networks. Genome Res. 13, 2498–2504. 10.1101/gr.1239303 14597658 PMC403769

[B56] Sharma A. Menche J. Huang C. C. Ort T. Zhou X. Kitsak M.. (2015). A disease module in the interactome explains disease heterogeneity, drug response and captures novel pathways and genes in asthma. Hum. Mol. Genet. 24, 3005–3020. 10.1093/hmg/ddv001 25586491 PMC4447811

[B57] Shi X. Barnes R. O. Chen L. Shajahan-Haq A. N. Hilakivi-Clarke L. Clarke R.. (2015). BMRF-Net: a software tool for identification of protein interaction subnetworks by a bagging Markov random field-based method. Bioinformatics 31, 2412–2414. 10.1093/bioinformatics/btv137 25755273 PMC4495295

[B58] Silberberg Y. Kupiec M. Sharan R. (2017). GLADIATOR: a global approach for elucidating disease modules. Genome Med. 9:48. 10.1186/s13073-017-0435-z 28549478 PMC5446740

[B59] Skov V. Knudsen S. Olesen M. Hansen M. L. Rasmussen L. M. (2012). Global gene expression profiling displays a network of dysregulated genes in non-atherosclerotic arterial tissue from patients with type 2 diabetes. Cardiovasc. Diabetol. 11:15. 10.1186/1475-2840-11-15 22340758 PMC3348024

[B60] Spirin V. Mirny L. A. (2003). Protein complexes and functional modules in molecular networks. Proc. Natl. Acad. Sci. U.S.A. 100, 12123–12128. 10.1073/pnas.2032324100 14517352 PMC218723

[B61] Staiger C. Cadot S. Györffy B. Wessels L. F. Klau G. W. (2013). Current composite-feature classification methods do not outperform simple single-genes classifiers in breast cancer prognosis. Front. Genet. 4:289 10.3389/fgene.2013.00289 24391662 PMC3870302

[B62] Stelzl U. Worm U. Lalowski M. Haenig C. Brembeck F. H. Goehler H.. (2005). A human protein-protein interaction network: a resource for annotating the proteome. Cell 122, 957–968. 10.1016/j.cell.2005.08.029 16169070

[B63] Szklarczyk D. Franceschini A. Kuhn M. Simonovic M. Roth A. Minguez P.. (2010). The STRING database in 2011: functional interaction networks of proteins, globally integrated and scored. Nucleic Acids Res. 39(Suppl. 1), D561–D568. 10.1093/nar/gkq973 21045058 PMC3013807

[B64] Tan P. K. Downey T. J. Spitznagel E. L. Jr Xu P. Fu D. Dimitrov D. S.. (2003). Evaluation of gene expression measurements from commercial microarray platforms. Nucleic Acids Res. 31, 5676–5684. 14500831 10.1093/nar/gkg763 PMC206463

[B65] Tanay A. Sharan R. Kupiec M. Shamir R. (2004). Revealing modularity and organization in the yeast molecular network by integrated analysis of highly heterogeneous genomewide data. Proc. Natl. Acad. Sci. U.S.A. 101, 2981–2986. 10.1073/pnas.0308661100 14973197 PMC365731

[B66] Tomita E. Tanaka A. Takahashi H. (2006). The worst-case time complexity for generating all maximal cliques and computational experiments. Theor. Comput. Sci. 363, 28–42. 10.1016/j.tcs.2006.06.015

[B67] Ulitsky I. Shamir R. (2007). Identification of functional modules using network topology and high-throughput data. BMC Syst. Biol. 1:8. 10.1186/1752-0509-1-8 17408515 PMC1839897

[B68] Ulitsky I. Shamir R. (2009). Identifying functional modules using expression profiles and confidence-scored protein interactions. Bioinformatics 25, 1158–1164. 10.1093/bioinformatics/btp118 19297352

[B69] Vandin F. Upfal E. Raphael B. J. (2011). Algorithms for detecting significantly mutated pathways in cancer. J. Comput. Biol. 18, 507–522. 10.1089/cmb.2010.0265 21385051

[B70] Vinayagam A. Stelzl U. Foulle R. Plassmann S. Zenkner M. Timm J.. (2011). A directed protein interaction network for investigating intracellular signal transduction. Sci. Signal. 4:rs8. 10.1126/scisignal.2001699 21900206

[B71] Vlaic S. Conrad T. Tokarski-Schnelle C. Gustafsson M. Dahmen U. Guthke R.. (2018). ModuleDiscoverer: identification of regulatory modules in protein-protein interaction networks. Sci. Rep. 8:433. 10.1038/s41598-017-18370-2 29323246 PMC5764996

[B72] Wang E. T. Sandberg R. Luo S. Khrebtukova I. Zhang L. Mayr C.. (2008). Alternative isoform regulation in human tissue transcriptomes. Nature 456, 470–476. 10.1038/nature07509 18978772 PMC2593745

[B73] Wuchty S. Zhang A. Walling J. Ahn S. Li A. Quezado M.. (2010). Gene pathways and subnetworks distinguish between major glioma subtypes and elucidate potential underlying biology. J. Biomed. Inform. 43, 945–952. 10.1016/j.jbi.2010.08.011 20828632 PMC7241867

[B74] Yeger-Lotem E. Riva L. Su L. J. Gitler A. D. Cashikar A. G. King O. D.. (2009). Bridging high-throughput genetic and transcriptional data reveals cellular responses to alpha-synuclein toxicity. Nat. Genet. 41:316. 10.1038/ng.337 19234470 PMC2733244

[B75] Yi S. Lin S. Li Y. Zhao W. Mills G. B. Sahni N. (2017). Functional variomics and network perturbation: connecting genotype to phenotype in cancer. Nat. Rev. Genet. 18, 395–410. 10.1038/nrg.2017.8 28344341 PMC6020840

[B76] Yu H. Braun P. Yildirim M. A. Lemmens I. Venkatesan K. Sahalie J.. (2008). High-quality binary protein interaction map of the yeast interactome network. Science 322, 104–110. 10.1126/science.1158684 18719252 PMC2746753

[B77] Yuan X. Chen J. Lin Y. Li Y. Xu L. Chen L.. (2017). Network biomarkers constructed from gene expression and protein-protein interaction data for accurate prediction of leukemia. J. Cancer 8, 278–286. 10.7150/jca.17302 28243332 PMC5327377

[B78] Zhang K. X. Ouellette B. F. (2011). CAERUS: predicting cancer outcomes using relationship between protein structural information, protein networks, gene expression data, and mutation data. PLoS Comput. Biol. 7:e1001114. 10.1371/journal.pcbi.1001114 21483478 PMC3068924

[B79] Zhao J. Zhang X. S. Zhang S. (2014). Predicting cooperative drug effects through the quantitative cellular profiling of response to individual drugs. CPT Pharmacometrics Syst. Pharmacol. 3:e102. 10.1038/psp.2013.79 24573337 PMC3944117

