# Supplementary material for: A Comprehensive Survey of Tools and Software for Active Subnetwork Identification
Source: Front Genet. 2019 Mar 5;10:155. doi: 10.3389/fgene.2019.00155 (PMC6411791; doi:10.3389/fgene.2019.00155)
Supplement: Supplementary file 1 [file Data_Sheet_1.pdf]

Supplementary Material: A comprehensive survey for active subnetwork identification

1 METHOD DESCRIPTIONS

1.1 DIAMOnD

DIAMOnD (DIseAse MOdule Detection) aims to identify the full disease module from the inputs consisting of a set of known disease proteins and an interaction protein network. The algorithm is tested with 70 diseases on a combined network of $13,460$ proteins from different datasets. The algorithm starts with marking input protein as seed nodes for an iterative procedure that expands those seed nodes across the entire network. The iteration first computes the *connectivity significance* p-value for every protein that has a connection to any protein in the seed nodes. The *connectivity significance* represents the probability that a protein has more connections to seed proteins than expected under the null hypothesis. The iteration then adds the protein that has the most significant p-value to the seed set and restarts the expanding procedure. The iteration can be continued until the module expands across the entire network or after a certain number of proteins are added to the seed set. The final modules obtained from the expanding procedure are the subnetworks that contain significant candidates for disease proteins.

The python script for DIAMOnD can be obtained from the author's Github repository. Currently, DIAMOnD is compatible with Python 2. To conduct network analysis using DIAMOnD, users need to provide information about the network in sparse adjacent matrix format as well as a list of genes associated with the phenotype of interest. The output includes the nodes of the subnetwork.

1.2 GXNA

The input of GXNA (Gene eXpression Network Analysis) includes an expression matrix and an interaction network. GXNA first normalizes the expression values and calculates the M-values using variance-stabilizing transform (Huber et al., 2002). It then selects genes with high variability in M-values (minimum standard deviation of 0.5) and with at least one interaction in the input network. GXNA calculates the score for each gene as t-statistic and the aggregate score of a subnetwork as the average t-statistics of the genes. In order to find interacting genes with high scores, GXNA provides two approaches: i) selecting high-scoring groups from pre-defined groups, and ii) searching for high-scoring sets from all possible subsets. In the first approach, one can rank and select pathways from databases such as KEGG, which consists of hundreds of pathways. However, many pathways are large (many genes) and not useful in identifying changes that affect only a small subnetwork. In addition, this approach will miss pathways not included in the database. To overcome the described limitations, GXNA looks at the local neighborhood of nodes in the network. Given a seed x and a positive integer r, the first approach searches for the set of all nodes that are connected to x by a path with at most r edges. In the second approach, the algorithm uses a greedy algorithm to expand the subgraph from a set of seed by selecting nodes that maximize the score of the subgraph. The initial seeds are selected so that they have a certain degree and are far from one another. The search process stops after the subgraph has reached a pre-defined size or adding the extra node decreases the score of the current graph. To calculate the significance of the discovered subnetworks,

GXNA permutes the sample labels to construct the null distribution of scores for each subgraph and compares the real score of the subgraph against the null.

GXNA source code written in C++ can be downloaded from author's website. It also needs to be compiled before running. The input for GXNA includes expression, phenotype and annotation data. To make the preparation for these required inputs more convenient, the author provides instruction on how to generate input files using R at `http://statweb.stanford.edu/~serban/gxna/R2GXNA.txt`. GXNA comes with two interaction graphs for human and mouse, which limits its use to these species.

1.3 MATISSE

The input of MATISSE (abbreviation of Module Analysis via Topology of Interactions and Similarity SEts) includes an expression matrix and a gene or protein network. The goal of the algorithm is to find a Jointly Active Connected Subnetworks (JACSs) of genes or proteins that share high-similarity. MATISSE first calculates the similarity between nodes using the Pearson correlation of gene expression and then computes the weight for each connecting edge using the similarity. The score of a subnetwork is calculated as the total edge weights. The JACS finding algorithm consists of three steps: (1) detection of relatively small, high-scoring gene sets (or seeds), (2) seed improvement, and (3) significance-based filtering. There are three methods for the identification of seeds: best-neighbors, all-neighbors and heaviest-subnet. In best-neighbors, the nodes are ranked based on their weighted degrees (the total weight of outgoing edges). The seed generating step picks the highest ranking node v and selects a set of $k-1$ neighbors (k is pre-defined) that maximizes the seed score. The algorithm repeatedly creates a seed and removes its nodes from the graph in order to generate many candidate seeds. All-neighbors algorithm is similar to best-neighbors, but adding all neighbors of v to the seed instead of selecting $k-1$ neighbors. In the heaviest-subnet method, a node with the smallest weighted degree is repeatedly removed until none remains. The highest-scoring, size-constrained subgraph that was encountered is selected as the seed. The subgraph is removed from the graph and search for the next seed begins. Seed improvement is done by applying a greedy algorithm to all the seeds simultaneously. The algorithm takes disjoint subnetworks and applies four moves in each iteration: (i) addition of an unassigned node to JACS, (ii) remove a node from JACS (iii) exchange a node between JACSs, and (iv) merge two JACSs. In every move, it increases the overall score of current subgraph and maintains connectivity of JACS. After a collection of JACSs is obtained, MATISSE calculates the p-value for each JACS by comparing its score against the null distribution of scores for random subnetwork of the same size. Only those JACS which have p-values below certain threshold pass the filtering stage.

1.4 CEZANNE

CEZANNE (Co-Expression Zone ANalysis using NEtworks) is a method that makes use of the probabilities (confident scores) for interactions (network edges) to find significant modules from the given networks. CEZANNE is built on top of MATISSE (Supplemental Section 1.3). The input of CEZANNE includes gene expression profiles and a protein-protein interaction (PPI) network with the interaction confidence information. The network confidence values can be obtained by using Purification Enrichment (PE) scores developed by (Collins et al., 2007). The CEZANNE framework consists of three steps: (i) seed identification, (ii) optimization, and (iii) significance filtering. In the seed identification process, CEZANNE first filter the input networks with edges that have the confidence greater than a certain threshold, and execute MATISSE on the unweighted filtered network with the input gene expression profiles to find initial seeds. The network is then weighed by assigning the interaction confidence score to each edge and recursively compute the minimum cut to find the q-connected seeds, where q is the probability that at least one edge connects a node from a subset of vertexes to another node that does not belong to that

subset. This process will stop when the weight of the minimum cut exceeds $T = -\log(1-q)$. In the optimization process, a greedy algorithm similar to MATISSE is used to optimize the initial seeds while maintaining the q-connectivity. The algorithm takes into account the weight edges such that no operation causes the minimum cut in a module to drop below T. In the significance filtering process, CEZANNE randomly shuffles the expression pattern of each gene then re-runs the algorithm for 100 times and the highest co-expression score obtained in each run was recorded. The empirical P-value for modules are then calculated according to the distribution of these recorded scores. Only modules with p-value smaller than 0.1 are retained.

MATISSE can be downloaded from the author's website. This Java package is ready to use without installation. The input includes an expression matrix and an interaction network. Sample networks for human and S. cerevisiae are provided on the author's website. MATISSE accepts interaction network files in other formats such as SIF (used by Cytoscape), BioGRID, KEGG or Entrez Gene. It also supports multiple ways of importing expression matrix into the program.

1.5 PinnacleZ

PinnacleZ takes an expression matrix and a protein network as input. It defines a subnetwork as a gene set that induces a single connected component in the network. The algorithm starts by standardizing the expression values for each gene (i.e., the expression values for each gene have mean 0 and standard deviation 1). For a particular subnetwork, PinnacleZ calculates the average z-score for each sample and then computes the subnetwork score as the mutual information between the resulted scores and the phenotype vector (a vector of 1s and 2s that represent diseases and controls, respectively). To construct a subnetwork, PinnacleZ starts with a random seed and iteratively expands the subnetwork with a node that is within a certain distance from the seed and yields the maximal score increase. The search stops when no addition increases the score over a specified improvement rate. PinnacleZ collects all of these modules together which are called real modules. To assess the significance of the identified subnetworks, PinnacleZ performs 3 tests of significance. The first test permutes the gene labels and repeatedly performs the search procedure to construct a "global" null distribution of all random subnetwork scores. The p-value of each real subnetwork is calculated by comparing the subnetwork's score against the global null distribution. The second test compares the score of the subnetwork against those of random subnetworks initialized from the same seed. The third test permutes the sample labels instead of gene labels. For each subnetwork, the real score is compared against those obtained from sample permutation. A subnetwork is significant if its p-value is significant in all of the three tests.

PinnacleZ was recently removed from Cytoscape app store due to its incompatibility with Cytoscape 3. However, users who want to conduct experiment with PinnacleZ on Cytoscape 2 can still download the plugin using the archive from the link `https://web.archive.org/web/20120105141450/http://chianti.ucsd.edu/cyto_web/plugins/pluginjardownload.php?id=170`. PinnacleZ requires users to input a gene expression matrix file in a simple tabular format, a condition classification file, and a network which can be easily imported or downloaded using Cytoscape built-in function. The resulted network can be visualized and explored using Cytoscape functions.

1.6 RME Module detection

The method proposed by (Miller et al., 2011) aims to identify functional modules using patterns of recurrent and mutually exclusive aberrations (RME patterns). The input includes a two-dimensional binary mutation matrix X in which rows represent samples and columns represent genes. In the input matrix X, if

sample i contains an alteration in gene j, then $X_{ij} = 1$, otherwise $X_{ij} = 0$. The algorithm starts with a filtering step to keep only genes that meet a set frequency of recurrence. The next step is to calculate edge weight, defined as the number of samples where exactly one of the pair is mutated divided by the number of samples where at least one of the pair is mutated. The output scores are filtered to keep only high-scoring values. The algorithm then constructs a global network with nodes and genes in the input matrix. The weighted edges are added corresponding to the filtered exclusivity scores. To be more specific, the scoring process applies Winnow (Littlestone, 1988) algorithm - a technique for learning a linear classifier from labeled examples, to score the exclusivity. The Winnow algorithm uses one gene as a classifier and the rest of the mutation array as training data. The output score represents how well each aberration in the classifier is predictive of non-aberration in each gene and vice versa. In the filtering process, all scores that higher or equal to the second highest score are retained.

After the global network is constructed, the algorithm uses each gene as a starting point in a combination of greedy and local search to find RME modules by improving the overall score but keeping the size below a certain limit. It then estimates the statistical significance of each discovered module using an algorithmic significance test described in (Milosavljević and Jurka, 1993). The algorithmic significance calculates the probability of the difference between bits length required to encode the binary matrix X by the discovered modules to the bits length required to encode the matrix under the null hypothesis. The module with the largest size and highest significance is retained.

RME Module Detection scripts can be download from author's website. Ruby language execution environment needs to be installed to run the provided scripts. In order to run the script, users must provide a binary matrix with detail about aberrant genes in each sample. No interaction network is needed. The script is straightforward and easy to use, however, it lacks utilities such as visualization tool.

1.7 BMRF-Net

The input of BMRF includes gene expression data and a protein interaction network. BMRF-Net is a method based on bagging Markov random field (BMRF) and simulated annealing search algorithm to find confident subnetworks from inputs consisting of a protein-protein interaction network and gene expression profiles. By using Markov random field, the method assumes that the score of one gene depends only on its neighbors in the network. The method first calculates the p-value for each gene (using t-test) and then convert it to z-score, which is named observed discriminative score. The method uses Gibbs distribution to compute the joint probability of the Markov random field of the network, and then the final estimated discriminative score is computed by a maximum a posteriori probability estimation which maximizes the likelihood of the posterior probability of the joint probability given the observed discriminative scores. A search algorithm based on simulated annealing is then applied to find a subnetwork with the maximum score from a given seed node. The score of the network is defined as the negative of the posterior function in terms of the estimated discriminative scores given the observed discriminative scores. In addition to stopping criteria of the simulated annealing algorithm, the annealing process will also stop when the network remains unchanged for 500 steps. BMFR-Net also performs a significance test using bootstrap to assess the statistical significance of the identified networks.

The java package can be obtained from SourceForge using the link provided in the main text. Currently, BMRF-Net only works on Unix environment. The required inputs for BMRF-Net are expression data, the label of samples, list of starting gene seeds and protein-protein interaction data. PPI data can be in a two-column matrix format and each row is a pair of proteins with interaction. BMRF-Net can also link to Cytoscape for visualization of the generated result.

1.8 COSINE

COSINE (COndition SpecIfic sub-NEtwork) takes condition-associated (tissue types, normal versus diseased samples, etc.) gene expression data and an interaction network as inputs. The software can handle multiple conditions. It calculates a score for each gene and each edge using F-statistic and Expected Conditional F-statistic (ECF-statistic) (Lai et al., 2004), respectively. The scores of edge and node are then standardized against the whole pool of edges and nodes, respectively. The score of a subnetwork is formulated as a weighted average of nodes and edges belonging to the subnetwork. COSINE defines the subnetwork extraction as a global optimization problem of finding a binary vector of length p (total number of genes) where the ith in the vector being 1 corresponds to this gene being included in the subnetwork, and 0 otherwise. The algorithm uses the genetic algorithm to search for the highest scoring subnetwork.

COSINE is an R package and is available on CRAN repository, which makes it easy to install and incorporate COSINE into data processing. To conduct analysis using COSINE, users need to provide expression data in different conditions as well as protein-protein interaction data in a two-column matrix format (a pair of proteins that interact with each other).

1.9 GLADIATOR

GLADIATOR (GLobal Approach for DIsease AssociaTed mOdule Reconstruction) aims to simultaneously identify disease modules for multiple diseases in a protein-protein interaction network. GLADIATOR takes inputs of multiple diseases along with its associated proteins and a PPI network with phenotype information. The algorithm first calculates the largest connected component for each disease in the network and use these components as the starting point (seed set) for the expansion of connected modules.

GLADIATOR makes use of the simulated annealing algorithm to traverse and expand the network. The goal of this process is to optimize the correlation between the phenotypic- and module-based disease similarities. The phenotypic-base similarity between two modules is calculated using the cosine similarity between the vectors of disease-associated phenotypes. The module-base similarity between two modules is calculated by using the Jaccard index. The annealing process is considered optimal when the objective function is minimal, which is the sum of the squared distance between the two similarities of each module. In the annealing step, a random module is selected, and either a random protein from the set of neighbors available for the current module is added, or a random non-seed protein from the current module is removed (proteins disconnected from the seed set caused by the removal will be also removed). The algorithm then recalculates the objective function and accept the last move if only it satisfies the algorithm's acceptance probability function. After the annealing process, the connected module expanded from the largest connected component of that disease is the resulting subnetwork for each disease.

GLADIATOR provides a simple Python script to perform the analysis. To start the analysis, users only need to provide a network file in a tabular format containing interactions and its types, and another file containing seed genes for different diseases. The output file resulted from the algorithm presents proteins module for each disease.

1.10 jActiveModules

The inputs for jActiveModules include a gene network and an expression matrix, in which rows represent genes and the columns represent samples. jActiveModule first calculates a p-value for each gene (by comparing diseases versus controls) and then converts the p-values to z-scores. It calculates the aggregate

score of a subnetwork (gene set) as the average score of the genes in the subnetwork. In order to properly capture the connection between expression and network topology, jActiveModule computes the z-scores of random gene sets to create a null distribution using a Monte Carlo approach. It then adjusts the scores of the underlying subnetworks using the mean and standard deviation of the null distribution. Finally, jActiveModules searches for high-scoring subnetworks using a simulated annealing approach. It initializes a subnetwork by assigning each node as either active or inactive with the probability of $\frac{1}{2}$. At each iteration, it randomly chooses a node and toggles the node's state (from active to inactive and vice versa). It then recalculates the aggregate score of the subnetwork. If the new score is greater than the old score, the state of the node is kept toggled. Otherwise, the node is kept toggled with a certain probability. The algorithm returns the high-scoring subgraph after a pre-defined number of iterations. jActiveModules allows users to search for M number of subnetworks simultaneously where M is another user-definable parameter. In the case of multiple conditions, z-scores are calculated for each condition (versus controls). The maximum value of the aggregate scores is considered the score of the subnetwork.

jActiveModule is a Cytoscape plugin, therefore, users need to first install Cytoscape. Afterward, users can easily install jActiveModule from the Cytoscape App Store. The input includes a network and a corresponding scoring data file to perform active subnetwork identification. The network can be easily imported from the predefined networks within the Cytoscape app. The network can also be downloaded from other available sources such as ndexbio.org and then imported to the app. jActiveModules provides a friendly and straightforward interface to adjust the algorithms. Thanks to the Cytoscape app, users can also easily visualize the data and explore the network in a very handy way.

1.11 MOEA

MOEA (Multi-Objective Evolutionary Algorithm) aims to find biologically meaningful modules in large-scale networks by solving a multi-objective problem which maximizes the network score and the prior knowledge contained in the active module. MOEA also takes inputs consisting of (i) gene expression profile to score the network and (ii) pathway knowledge to measure the enrichment of functional groups from discovered modules. From the gene expression profile, MOEA uses a method similar to the BioNet's method (Beisser et al., 2010) to calculate gene scores and network scores. MOEA then uses a non-dominated sorting genetic algorithm proposed by (Deb et al., 2002) to solve the network optimization problem with multiple objectives including maximizing the network score and maximizing the coverage of the current module on its mapped KEGG pathway. In addition to the two objectives, MOEA uses the algebraic connectivity (the second-smallest eigenvalue of the Laplacian matrix) of the network to ensure the connectedness of the network in the optimization process.

MOEA is delivered as a bundle of Matlab scripts. However, the software is poorly documented, which can make it hard for users who are not familiar with Matlab to perform the analysis. To be able to run the main analysis using a genetic algorithm, users need to format the data using a provided script for data preparation. Users need to provide a p-value file generated from gene expression matrix, a network file in a triple columns format to construct the connectivity matrix of the network, and a gene-pathway mapping file indicating the relationship between genes and pathway. Afterward, users can use the main script to perform the analysis. The final result will contain a Pareto front of gene sets.

1.12 BioNet & Heinz

The BioNet package provides a set of tools to integrate gene expression analysis into biological networks analysis. The Heinz tool on another hand, process maximal-scoring process to extract subgraphs from

the input network. The input for the subnetwork analysis using BioNet and Heinz include an interaction network and a set of p-values for each gene in that network. Those p-values can be gathered by performing multiple analyses including differential expression analysis or through survival analysis. In the first step of the subnetwork analysis, BioNet aggregate multiple p-values for a gene into one single p-value by using uniform order statistics. The new set of p-values obtains from all genes in the network then be fitted in a beta-uniform mixture model to calculate the estimated maximum-likelihood parameters which are used to score the statistical significance of differential expression of the gene. The obtained scores are then adjusted to control the estimated upper bound of the false discovery rate. The scored network is then passed to $heinz$ to find the maximum-weight subgraph. $Heinz$ turns the maximum-weight connected subgraph problem into the Prize-Collecting Steiner Tree (PCST) problem by subtracting the score of each node to the minimum score of all nodes in the network ($minScore$). The scores of nodes are now positive and the cost of each edge is now equal to $-minScore$. $Heinz$ makes use of the algorithm proposed by (Ljubić et al., 2006) to find the subgraph for the PCST problem. The algorithm starts by converting the undirected input network into a directed network and add an artificial root into the network. Integer linear programming with a number of linear inequalities to constrain the solution is applied to the transformed directed network. The result from this step is one optimal subnetwork. $Heinz$ allows users to explore different sub-optimal network by adding a hamming distance to the optimal subnetwork to constrain the different of the returned sub-optimal networks.

BioNet is an R package can be easily installed from Bioconductor repository. Although Heinz takes part in subnetwork identification analysis with BioNet, Heinz is a python software separately distributed and can be downloaded from the author website. The network can be easily loaded using BioNet from a common network SIF file type or tabular file. After calculating the network score using expression data, users need to export the network to work with Heinz software. The result from Heinz can be loaded back into BioNet session and visualized using Cytoscape. Although the analysis with BioNet and Heinz is straightforward, users have to switch back and forth between the R environment and the command line interface because the two packages are written in two different programming languages.

1.13 HotNet & HotNet2

The input of HotNet includes an interaction network and the score for each gene that represent its mutation frequency across samples. HotNet makes use of heat diffusion algorithm to identify subnetworks that are mutated more than expected. To measure how genes in the network impact each other, HotNet use diffusion following the interaction between genes to distribute the heat from one gene to its neighbors. In each step of the diffusion process, each gene will pass or receive heat from its neighbors until the process is equilibrium. After the diffusion process, HotNet gathers subnetworks by selecting only gene relationships (which represents how much heat were distributed through the edge linking two genes) above a certain threshold. Finally, HotNet evaluates statistical significance (p-values and False Discovery Rate) of an obtained subnetwork by comparing the module heat score against score distribution obtained from the random permutation of mutation data. HotNet2 extends HotNet in multiple directions. Major differences between two versions including (i) HotNet2 uses insulated heat diffusion which retains some of the genes' own heat and diffuses the leftover heat rather than diffuses all heat to its neighbors, (ii) HotNet2 takes into account the direction of heat traveled in which the influences of heat traveled between two nodes on each node are different. Both HotNet and HotNet2 return a set of subnetworks with p-values and FDR-adjusted p-values.

The provided Python scripts for the software can be easily downloaded and install from its Github page. To start the analysis, users need to provides several data files in different formats to generate heat score files and network files. The heat scores can be generated from mutation data or from several other formats. The network files can also be generated from tabular nodes and edges list. The subnetwork is then identified using those files and can be visualized through an interactive web application provided by the software.

1.14 RegMOD

The input of RegMOD includes an expression matrix and an interaction network. RegMOD first calculates edge weights using Pearson's correlation and the score for each gene using the signal to noise ratio (SNR), i.e., mean difference (between disease and control samples) divided by the sum of standard deviations. It then smooths out outlier gene scores and estimates the underlying active score by integrating edge weights and network topology of genes. It uses support vector regression (Vapnik, 1998) with diffusion kernel (Kondor and Lafferty, 2002) to infer each gene's underlying active score f_i. This procedure can eliminate acute changes among neighboring genes in the network and infer underlying scores of genes whose expression level could not be measured. A gene with low SNR score but functioning as a bridge to connect high weighted genes will get a new high f score. Conversely, a gene with high SNR but interacting with low score genes will get a new low f score. Finally, genes with score less than $\bar{f} - \theta\sigma$ ($\bar{f}$ is the median, σ is the median standard deviation, and $\theta = 6$ by default) are considered down-regulated while those with score less than $\bar{f} + \theta\sigma$ are considered up-regulated. The corresponding subnetworks are the up- and down-regulated modules.

RegMOD is distributed as a set of Matlab scripts. The analysis with RegMOD is simple and straightforward. Users only need to provide an adjacent matrix indicating connectivities in the network, and a list of scoring nodes. The output will be the predicted active score for each node.

1.15 ResponseNet

The input of ResponseNet includes a network and a weighted list of stimulus-related proteins and differentially expressed (DE) genes. Higher weights increase the probability that the corresponding genes/proteins will appear in the output subnetwork. ResponseNet designs the graphical model as follows: i) proteins and genes are represented as separate nodes, ii) directed edge leads from a protein to a gene only if they correspond to a transcription factor and its target gene, and iii) each edge is associated with a likelihood probability. To find paths of high probability from source nodes (stimulus-related proteins) to sink nodes (stimulus-related DE genes), ResponseNet implements a "flow algorithm" that is well-known for efficiently finding connectivity in graphs (Cormen et al., 2009). Flow algorithms deliver an abstract flow from a source (proteins) to a sink (genes) through the pipes (edges) for which the capacity are represented by the likelihood probability. ResponseNet utilizes linear programming tools provided by LOQO (Vanderbei, 1999) to maximize the flow (i.e., to include as many sources and sinks as possible) while minimizing the cost (i.e., to decrease negative log of likelihood probability). ResponseNet outputs the signaling and regulatory subnetwork by which stimulus-related proteins detected by genetic screens may lead to the measured transcriptomic response.

ResponseNet provides a simple web-based application to run the algorithm at http://netbio.bgu.ac.il/respnet. It currently only supports two organisms: human and yeast. The application requires input a source set file and a target set file in a specific format. Users can also visualize and interact with the resulted subnetwork.

1.16 TimeXNet

The input of TimeXNet consists of i) a weighted interaction network, ii) three lists of DE genes during three consecutive intervals (initial, intermediate, and late stage) on exposing the cell to the external stimulus, and iii) gene scores or log fold change. Using the scores of the genes and the reliability weights of interactions in the input molecular network, TimeXNet aims to identify the most probable paths within the interaction network connecting the initial response genes to the late effectors while incorporating the intermediate regulators. TimeXNet uses the minimum-cost flow optimization algorithm (Patil et al., 2013) to identify such paths in the network. In order to solve the optimization problem, TimeXNet makes use of the GNU Linear Programming Kit (GLPK). Finally, statistical significance is calculated for each gene in the discovered optimal subnetwork by a re-sampling procedure. The procedure re-runs the analysis with random source and target nodes to obtain random modules. The p-value is calculated as the fraction of solutions in which a gene was identified with an equal or higher flow than that in the optimal subnetwork and with at least all the connecting edges in the optimal subnetwork. The output is the optimal sub-network with its statistical significance evaluation for each gene.

TimeXNet is available both as web interface and software package. The web interface can be accessed at http://txnet.hgc.jp. The software package can be easily downloaded and installed from the software website. The Java Runtime Environment and GNU Linear Programming Kit are also required to installed to run the software. TimeXNet provides an intuitive and simple interface. It requires users to input specific format data including log fold change data, data files for gene/protein in different stages group by time (source, intermediate, and late), and the network file. For the web-based application, the network can also be selected from the HitPredict database. TimeXNet also provides simple visualization function to interact with network data.

1.17 EnrichNet

EnrichNet is a web-interface application providing network-based enrichment analysis method to identify the functional relations between the interesting genes or proteins and cellular pathways. EnrichNet requires three input parameters including i) 10 or more human genes or proteins, ii) the selection of provided pathway databases from which reference genes/proteins will be extracted, and iii) a molecular network (as default, EnrichNet use a human interactome graph from STRING 9.0 database with edges weighted by STRING combined confidence score normalized to range $[0, 1]$).

The analysis starts with mapping the input gene/protein set (target set) and reference datasets onto the input molecular network. EnrichNet then scores the distance between the target set to all references datasets using the random walk with restart algorithm proposed by (Yin et al., 2010). In short, the random walk procedure starts with equal probability from each nodes corresponding to the target set and randomly choose its neighbors over time. The random walk with restart algorithm allows the procedure to restart the walk at the source nodes with a pre-defined probability p (here $p = 0.9$). After the walk, a distance score is obtained for each of the genes in the reference pathways, which results in a distance score vector for each pathway. Another distance vector score representing the average distance between the target set and a background model containing all reference pathways is then calculated. A final Xd-score for each reference pathways is measured by the weighted averaging of the difference between the two distance scores in a discretized distribution. The Xd-score measurement emphasizes the lower distances by assigning higher weights to smaller distances and lower to the higher distance. The Xd-scores are then compared against the classical over-representation scores for overlapping datasets computed using Fisher's exact test to generate

a regression plot for users to choose a threshold for the Xd-scores. The pathways corresponding to filtered Xd-score can be visualized as subnetworks in the molecular interaction network on the EnrichNet webtool.

EnrichNet provides a user-friendly web interface. Users only need to input the gene/protein identifier to begin the analysis. Molecular network data and annotation database can be easily selected from the website. Users can also upload their version of the molecular network with a specific format required by the software.

1.18 Walktrap-GM

The input of Walktrap-GM includes a global network of biological interaction and gene expression data. The score of each node is calculated as the absolute log fold change while the weight of an edge is the square of the mean score of the two connected nodes. The cumulative activity of a module is a squared transformation of the average weighted expression for all nodes in the module; where the weight of a given node is the maximum fold change of probes corresponding to its gene symbol. To travel in the network, Walktrap-GM uses the random walk algorithm which transits from the current node to its adjacent nodes with a probability based on the weight of the linking edge and the degree of the current node. Using the transition probabilities, the distance between two nodes and between two communities formed by the random walk process are then calculated. The traverse will merge two communities if it minimizes the mean of the squared distances between each node and its community. The merge process will stop when it maximizes (i) the size, or (ii) the modularity (which represents the difference between the fraction of edges inside the community and the fraction of edges bound to the community), or (iii) the module score (a test statistic comparing the cumulative activity of a module against the bootstrap distribution).

Walktrap-GM is an R package and can be downloaded from the Github page of the software at https://github.com/petrochilos/walktrap-gm. To start the analysis, users need to provide a list of fold change values and also the molecular network. The molecular network has to be constructed as an igraph object (igraph is a library collection for creating and manipulating graphs and analyzing networks in R). After that, users can calculate the graph weights and score the communities using functions provided by the software.

1.19 MEMo

The goal of MEMo is to identify sets of connected genes that are recurrently altered, likely to belong to the same biological process, and exhibit patterns of mutually exclusive genetic alteration across multiple patients. The input includes somatic mutations, copy number alteration (CNA), and mRNA expression. In Step 1, MEMo select genes for which: i) the number of somatic mutation is significantly higher than expected by chance (using somatic mutation and binomial test), or ii) the genome is altered more frequently (using permutation test) and CNA is concordant with mRNA expression (i.e. amplification in CNA is supported by up-regulation). In Step 1, MEMo also builds a binary matrix M (genes are rows and samples are columns) indicating if the gene is significantly altered in a sample. In step 2, MEMo performs a global gene comparison test to determine all pairs of genes that are functionally connected. Given two nodes, MEMo calculates the ratio between the intersection and the union of the two node's neighbors. It then sets a threshold to determine if two genes are proximal. In Step 3, MEMo builds a graph of all similar gene pairs and then extract all maximal cliques, i.e., all fully connected subgraphs. In order to calculate the significance of each module, MEMo permutes M and calculates the p-value as the number of genomic alterations (for any gene in the clique) in random alteration matrices more extreme than in the actual M divided by the total number of permutations.

MEMo is delivered as a set of Python scripts and Java classes. Users first need to construct the database from a PPI network file in SIF file format, a tabular gene info file, and a pathway gene set information file from Broad Institute. To start the analysis, the software also requires multiple different files in different specific formats including mutation information file, copy number alteration file, copy number driven genes file, recurrently deleted/amplified regions of interest files, identifiers for all samples in the study, and a recurrently altered genes file. The application generates the reports for different discovered modules in web format with visualization supports.

1.20 ModuleDiscoverer

ModuleDiscoverer aims to detect regulatory modules from a protein-to-protein network (STRING database) and phenotype-associated gene expression data. The algorithm starts by transforming the network into an undirected labeled graph where each vertex can be labeled with more than one proteins (in such case, the proteins in the label form a clique). The weight of the edge connecting v_x and v_y denotes for the number of relations between proteins represented by v_x and proteins represented by v_y. Initially, all edges have weight 1. The algorithm then selects random seeds and randomly explores vertices to find minimal cliques of size three. It merges the vertices and updates the weights of the edges. The algorithm also attempts to iteratively enlarge the cliques until they cannot be enlarged further. Once a node becomes part of a clique, it cannot become part of another clique. In order to calculate the statistical significance of each clique, ModuleDiscoverer calculates the p-values and for each gene using the gene expression data and then selects a set of differently expressed genes (DEGs) using the significance cutoff of 0.05. It then calculates the score (p-value) for each clique using over-representation test of DEGs (ORA or Fisher's exact test). Next, it permutes the genes to construct a distribution of scores (p-values) for each clique. The final p-value of each clique is calculated by comparing the actual score against the null distribution. Based on a user-defined p-value, the algorithm filters the significantly enriched cliques and then merge all significant cliques into one large regulatory module.

ModuleDiscoverer is delivered as an R script file. Users need to source the provided R script file to make use of ModuleDiscoverer functions. To start the analysis, users need to construct an adjacency matrix indicating connectivity between each node in the network and a table of all vertices in the network containing the weight and degree of each vertex. ModuleDiscoverer provides a simple tutorial for users to start the analysis with the provided script. However, a non-packaged software for R makes it difficult for users to install and manage the functions.

1.21 ClustEx

The input of ClustEx includes an interaction network and gene expression. ClusterEx is a two-step method for identifying responsive gene modules. In the clustering step, ClustEx identifies DE genes, computes the distance between gene pairs, and then cluster the genes according to their distance in the gene networks using hierarchical clustering. Since similarity is often represented by correlation, ClustEx defines the distance between two connected genes as 1 minus correlation of gene expression. The distance between any two genes is defined as the shortest path between the genes using Dijkstra's algorithm. In the extending step, the intermediate genes on the k-shortest paths between the DE genes were added to form the final responsive gene modules. To reduce the false positives and computational cost for finding the k-shortest paths between all pairs of nodes in the whole gene network, the extending step was implemented as follows: first, it adds the genes on the shortest paths between the DE genes to form a connected subnetwork; then it extends the subnetwork by one step in the whole gene network; finally, it identifies the responsive gene modules by extracting all the genes and edges on the 10-shortest paths. In order to assess the significance

of the resulted subnetworks, ClustEx calculates edge score as the product of the two genes' standard deviation and correlation and subnetwork score as the sum of edge scores. The statistical significance of each subnetwork is computed by comparing the actual score against the score distribution obtained from random subnetworks of the same size.

ClustEx provides a binary executable for Linux and Windows. Users on other platform need to compile ClustEx from source code written in C++. ClustEx provides a simple command to perform the analysis. To get started, users need to construct a seed genes file and a network file in a simple tabular format and pass these file to ClustEx command. The output subnetwork can be easily visualized using Cytoscape application.

1.22 SAMBA

The input of SAMBA (Statistical-Algorithmic Method for Bicluster Analysis) includes interaction networks, gene expression, and transcriptome factor (TF) binding. SAMBA models all genomic information as a weighted bipartite graph where nodes on one side represent the genes and nodes on the other side represent properties of the protein encoded by them. A weighted edge connecting a gene with a property represents the probability of the gene having that property. Each edge connecting nodes in two sides is assigned a weight reflecting the probability of a gene node has the measurement in the connected property node. To search for statistically significant subgraphs, module seeds are formed by using biclustering to detect dense and heavy structure in the graph and then optimize them to locally optimal modules. The ultimate modules are obtained by removing modules that are similar to the highest-scoring module. Finally, SAMBA calculates the statistical significance value of discovered modules by comparing with randomly shuffled graphs.

Since SAMBA has been incorporated in Expander software suite, users need to install the Expander software suite to make use of the method. SAMBA requires users to input an expression matrix data as a tabular data file or CEL file, and a network in a SIF file format. The network file can also be easily downloaded from the software itself. The expander software suite also provides functions to convert and match gene id in different format between the network and the expression matrix. SAMBA method can be easily invoked as an option of the Expander software suite.

2 APPLICATIONS

Active subnetwork identification has been widely applied to real-world settings to find disease gene signatures, dysfunctional pathways, the relationship among diseases, etc. Among the 22 methods we present in this survey, many methods were utilized and took an important role in different studies. Among 22 methods, jActiveModules is one of the most widely used tools. As a Cytoscape plugin, jActiveModules takes its advantages in network visualization and manipulation. At the time of this survey, we found more than 70 studies made use of this plugin. Following jActiveModules, BioNET as an R package, was also used in more than 30 publications. Other tools including EnrichNet, MEMo, and MATISSE was used in over ten to 20 publications. For the rest of the list, we found less than ten applications that utilized it.

The following part of this section will present the list of publications in **DOI ID** we have found so far that made use of active subnetwork identification software listed on this survey.

jActiveModules: we found 77 manuscripts that applied jActiveModules in real-world application. The DOIs and PubMed IDs are as follows: 10.1016/j.gene.2014.03.022, 10.1016/j.bbrep.2018.01.001, 10.1093/bioinformatics/btw164, 10.1016/j.gene.2014.02.001, 10.1016/j.ajhg.2013.04.019,

10.1186/1475-2840-11-15, 10.1016/j.mito.2009.02.004, 10.1039/C5MB00622H, 10.7150/jca.17302, 10.1371/journal.pone.0181667, 10.1038/srep35228, 10.1080/19336896.2015.1019694, 10.1186/1755-8794-7-S1-S3, 10.1039/C0MB00325E, 10.5897/JMPR, 10.1371/journal.pone.0189886, 10.1016/j.gene.2013.08.059, 10.3389/fgene.2016.00200, 10.1039/b912078e, 10.1016/j.lfs.2008.07.019, 10.1093/toxsci/kfq086, 10.1007/s12263-015-0484-0, 10.1186/2050-6511-14-9, 10.1111/gbb.12190, 10.1002/tox.22338, 10.1097/MD.0000000000007673, 10.1007/s10142-016-0533-9, 10.1371/journal.pone.0010465, 10.1371/journal.pone.0106646, 10.1016/j.jmb.2009.08.077, 10.1177/0022034515581186, 10.1093/toxsci/kfu052, 10.1186/1752-0509-2-101, 10.1038/ncomms9234, 10.1002/art.37735, 10.1016/j.mrfmmm.2009.10.008, 10.1142/9789812701626_0033, 10.1093/toxsci/kft018, 10.1089/jir.2014.0150, 10.1093/toxsci/kfm226, 10.1016/j.devcel.2010.06.014, 10.1371/journal.pone.0013606, 10.1371/journal.pone.0081253, 10.1016/j.virusres.2011.08.015, 10.1074/jbc.M109.095570, 10.1038/s41598-017-07189-6, 10.1021/es1032579, 10.1371/journal.pgen.1007498, 10.1089/zeb.2014.0995, 10.1155/2016/4672841, 10.1186/s12974-017-0825-6, 10.1016/j.fsi.2015.04.003, 10.1007/s11829-017-9562-0, 10.1016/j.neuroscience.2018.03.033, 10.1007/s10886-018-0972-y, 10.1534/g3.113.009936, 10.1038/srep39297, 10.1167/tvst.6.4.12, 10.1371/journal.pone.0093626, 10.1111/gbb.12190, 10.1038/ncomms5649, 10.3389/fimmu.2018.01241, 10.1038/mp.2012.163, 10.1007/s00438-013-0743-y, 10.1074/mcp.M112.024851, 10.18257/raccefyn.364, 10.1371/journal.pone.0172427, 10.1016/j.taap.2018.09.010, 10.1093/toxsci/kfu052, 10.1186/gb-2007-8-5-r70, 10.1371/journal.pgen.1002699, 10.1098/rspb.2013.1381, 10.1111/febs.12705, 10.1038/s41598-017-17505-9, 10.1038/srep25131, 10.1105/tpc.110.082529, 10.1371/journal.pgen.1002699.

PinnacleZ: we found 5 manuscripts that applied PinnacleZ in real-world application. The DOIs and PubMed IDs are as follows: 10.1155/2013/210253, 10.1016/j.jbi.2010.08.011, 10.1093/bioinformatics/btr533, 10.1523/JNEUROSCI.3180-09.2009, 10.1371/journal.pone.0034796

DIAMOnD: we found 8 manuscripts that applied DIAMOnD in real-world application. The DOIs and PubMed IDs are as follows: 10.1038/srep27414, 10.1101/335679, 10.1093/hmg/ddv001, 10.18632/oncotarget.22048, 10.1101/383588, 10.1371/journal.pcbi.1005522, 10.7554/eLife.34423.001, 10.7554/eLife.39188.

GXNA: we found 5 manuscripts that applied GXNA in real-world application. The DOIs and PubMed IDs are as follows: 10.1158/0008-5472.CAN-16-1959, 10.1039/b912078e, 10.5220/0004915202650270, 10.1371/journal.pone.0082460, 10.4236/jilsa.2014.64012.

MATISSE: we found 15 manuscripts that applied MATISSE in real-world application. The DOIs and PubMed IDs are as follows: 10.1186/1752-0509-5-148, 10.1073/pnas.1115753108, 10.1089/omi.2013.0050, 10.1105/tpc.112.104513, 10.1016/j.ajpath.2012.03.043, 10.1371/journal.pone.0070498, 10.1101/gr.100594.109, 10.1038/mp.2016.109, 10.1016/j.bbrc.2010.01.101, 10.1371/journal.pgen.1002309, 10.1111/ppl.12326, 10.1038/msb.2008.42, 10.1504/IJDMB.2015.067320, 10.1186/1752-0509-5-148, 10.1158/1538-7445.

BioNET: we found 35 manuscripts that applied BioNET in real-world application. The DOIs and PubMed IDs are as follows: 10.3892/ol.2015.4042, 10.3892/ol.2016.5497, 10.1186/1755-8794-5-27, 10.1700/1778.19294, 10.3892/mmr.2018.9300, 10.1016/j.mad.2015.05.008, 10.3892/mmr.2017.7157, 10.3892/ol.2017.7310, 10.3892/ol.2016.4663, 10.1038/psp.2013.79, 10.3892/ol.2018.8615, 10.1186/s13018-015-0275-8, 10.1016/j.prp.2017.01.019, 10.1038/labinvest.2014.3, 10.1371/journal.pone.0147475, 10.4137/EBO.S8540, 10.1111/j.2047-2927.2013.00122.x, 10.3892/mmr.2017.6800, 10.1016/j.immuni.2015.02.005, 10.1371/journal.pone.0095340,

10.1371/journal.pcbi.1004293, 10.1007/s00335-014-9536-9, 10.1038/nmicrobiol.2016.180, 10.1007/s00125-016-4182-2, 10.1038/srep35436, 10.1038/srep00515, 10.1186/1471-2164-14-483, 10.1186/s12918-017-0388-2, 10.1371/journal.pcbi.1002227, 10.1186/s12964-015-0106-x, 10.1371/journal.pone.0168183, 10.4049/jimmunol.1800161, 10.1371/journal.pone.0040498, 10.1093/hmg/ddx036, 10.1016/j.celrep.2016.06.038.

HOTNET: we found 6 manuscripts that applied HOTNET in real-world application. The DOIs and PubMed IDs are as follows: 10.1038/cr.2017.146, 10.18632/oncotarget.23719, 10.1038/ncomms4156, 10.1038/nature12222, 10.1371/journal.pcbi.1002820, 10.1056/NEJMoa1301689.

ResponseNET: we found 2 manuscripts that applied ResponseNET in real-world application. The DOIs and PubMed IDs are as follows: 10.1093/nar/gky618, 10.1093/brain/awy045.

EnrichNet: we found 21 manuscripts that applied EnrichNet in real-world application. The DOIs and PubMed IDs are as follows: 10.3892/mmr.2015.3583, 10.1371/journal.pone.0156006, 10.1016/j.nbd.2014.11.002, 10.1155/2015/872983, 10.3233/JPD-150737, 10.1016/j.cbd.2018.04.004, 10.1186/s12953-017-0114-4, 10.5114/biolsport.2018.70746, 10.18632/oncotarget.13346, 10.1186/s12918-014-0107-1, 10.1007/s11033-016-4021-z, 10.1007/s00439-014-1473-x, 10.1371/journal.pone.0145696, 10.1371/journal.pone.0150239, 10.3389/fgene.2015.00288, 10.1371/journal.pgen.1006682, 10.1093/molbev/msw004, 10.1021/acschembio.7b00869, 10.3945/ajcn.117.156216, 10.1371/journal.pone.0178316, PMC5338275.

ModuleDiscoverer: we found 3 manuscripts that applied ModuleDiscoverer in real-world application. The DOIs and PubMed IDs are as follows: 10.7150/thno.24333, 10.1074/mcp.RA117.000069, 10.1186/s12918-018-0620-8.

ClustEx: we found 3 manuscripts that applied ClustEx in real-world application. The DOIs and PubMed IDs are as follows: 10.1093/bioinformatics/btr619, 10.1039/C4MB00329B, 10.1039/C2MB25065A.

MEMo: we found 13 manuscripts that applied MEMo in real-world application. The DOIs and PubMed IDs are as follows: 10.1186/1471-2105-14-29, 10.1038/nature21386, 10.3389/fcvm.2017.00036, 10.1038/nature11412, 10.1007/s10549-013-2699-3, 10.1016/j.celrep.2016.02.024, 10.1038/nature12222, 10.1007/s00438-018-1416-7, 10.1038/nature12113, 10.1016/j.cell.2015.09.033, 10.1084/jem.20132120, 10.1155/2013/206875, 10.1158/1078-0432.CCR-12-2093.

SAMBA: we found 3 manuscripts that applied SAMBA in real-world application. The DOIs and PubMed IDs are as follows: 10.1093/nar/gkl937, 10.1093/bioinformatics/btn034, 10.1186/1752-0509-3-59.

REFERENCES

Beisser, D., Klau, G. W., Dandekar, T., Muller, T., and Dittrich, M. T. (2010). BioNet: an R-Package for the functional analysis of biological networks. *Bioinformatics* 26, 1129–1130

Collins, S. R., Kemmeren, P., Zhao, X.-C., Greenblatt, J. F., Spencer, F., Holstege, F. C., et al. (2007). Toward a comprehensive atlas of the physical interactome of saccharomyces cerevisiae. *Molecular & Cellular Proteomics* 6, 439–450

Cormen, T. H., Leiserson, C. E., Rivest, R. L., and Stein, C. (2009). *Introduction to algorithms* (MIT press), 3rd edn.

Deb, K., Pratap, A., Agarwal, S., and Meyarivan, T. (2002). A fast and elitist multiobjective genetic algorithm: Nsga-ii. *IEEE transactions on evolutionary computation* 6, 182–197

Huber, W., Von Heydebreck, A., Sültmann, H., Poustka, A., and Vingron, M. (2002). Variance stabilization applied to microarray data calibration and to the quantification of differential expression. *Bioinformatics* 18, S96–S104

Kondor, R. I. and Lafferty, J. (2002). Diffusion kernels on graphs and other discrete structures. In *Proceedings of the 19th international conference on machine learning*. vol. 2002, 315–322

Lai, Y., Wu, B., Chen, L., and Zhao, H. (2004). A statistical method for identifying differential gene–gene co-expression patterns. *Bioinformatics* 20, 3146–3155

Littlestone, N. (1988). Learning quickly when irrelevant attributes abound: A new linear-threshold algorithm. *Machine learning* 2, 285–318

Ljubić, I., Weiskircher, R., Pferschy, U., Klau, G. W., Mutzel, P., and Fischetti, M. (2006). An algorithmic framework for the exact solution of the prize-collecting steiner tree problem. *Mathematical programming* 105, 427–449

Miller, C. A., Settle, S. H., Sulman, E. P., Aldape, K. D., and Milosavljevic, A. (2011). Discovering functional modules by identifying recurrent and mutually exclusive mutational patterns in tumors. *BMC Medical Genomics* 4, 34

Milosavljević, A. and Jurka, J. (1993). Discovering simple dna sequences by the algorithmic significance method. *Bioinformatics* 9, 407–411

Patil, A., Kumagai, Y., Liang, K.-c., Suzuki, Y., and Nakai, K. (2013). Linking transcriptional changes over time in stimulated dendritic cells to identify gene networks activated during the innate immune response. *PLoS computational biology* 9, e1003323

Vanderbei, R. J. (1999). Loqo user's manual—version 3.10. *Optimization methods and software* 11, 485–514

Vapnik, V. (1998). *Statistical learning theory. 1998*, vol. 3 (Wiley, New York)

Yin, Z., Gupta, M., Weninger, T., and Han, J. (2010). A unified framework for link recommendation using random walks. In *Advances in Social Networks Analysis and Mining (ASONAM), 2010 International Conference on* (IEEE), 152–159
